# Rough Set Approach to Incomplete Multiscale Information System

**DOI:** 10.1155/2014/538968

**Published:** 2014-08-17

**Authors:** Xibei Yang, Yong Qi, Dongjun Yu, Hualong Yu, Xiaoning Song, Jingyu Yang

**Affiliations:** ^1^School of Economics and Management, Nanjing University of Science and Technology, Nanjing 210094, China; ^2^School of Computer Science and Engineering, Jiangsu University of Science and Technology, Zhenjiang 212003, China; ^3^Key Laboratory of Intelligent Perception and Systems for High-Dimensional Information, Nanjing University of Science and Technology, Nanjing 210094, China

## Abstract

Multiscale information system is a new knowledge representation system for expressing the knowledge with different levels of granulations. In this paper, by considering the unknown values, which can be seen everywhere in real world applications, the incomplete multiscale information system is firstly investigated. The descriptor technique is employed to construct rough sets at different scales for analyzing the hierarchically structured data. The problem of unravelling decision rules at different scales is also addressed. Finally, the reduct descriptors are formulated to simplify decision rules, which can be derived from different scales. Some numerical examples are employed to substantiate the conceptual arguments.

## 1. Introduction

As one of the important mathematical tools for granular computing [1, 2], the theory of rough set [3] has been demonstrated to be useful in fields such as data mining, knowledge discovery, decision support, machine learning, and pattern recognition.

Pawlak's rough set was proposed on the basis of an indiscernibility relation, which can generate a granulation space on the universe of discourse. Such granulation space is actually a partition since the indiscernibility relation is an equivalence relation. With respect to different requirements, a variety of the expanded rough sets models have been proposed. For example, the tolerance relation [4–7], similarity relation [8–10], characteristic relation [11, 12], and neighborhood system [13] based rough sets can be used to deal with the incomplete information systems; the dominance-based rough set approach [14–18] can be used to deal with the multicriteria decision problems; the covering based rough sets [19–22] are constructed on the basis of a covering, which is an expansion of the partition on the universe; the fuzzy rough set approaches [23–26] are proposed to approximate the fuzzy concepts in the fuzzy environments; the variable precision rough sets approaches [27–29] allow some inconsistency to exist, which can not only solve classification problems with uncertain data but also relax the boundary definition of Pawlak's rough set to improve the suitability.

Obviously, the above rough sets are constructed on the basis of one and only one set of the information granules, which can be generated from a binary relation or a covering. From this point of view, we may call these rough sets the single-granulation rough sets. In single-granulation rough sets, a partition is a granulation space, a binary neighborhood system induced by a binary relation is a granulation space, and a covering is also a granulation space. Nevertheless, it should be noticed that, in [30], the authors said that we often need to describe concurrently a target concept from some independent environments; that is, multigranulation spaces are needed in problem solving. From this point of view, Qian et al. [31–33] proposed the concept of the multigranulation rough sets. The main difference between single-granulation and multigranulation rough sets is that we can use multidifferent sets of the information granules for the approximating of target concept. Since each set of the information granules can be considered as a granulation space, then the space induced by multidifferent sets of the information granules is referred to as the multigranulation space. For example, a family of the partitions can be regarded as a partitions based multigranulation space.

Presently, the development of multigranulation rough sets approaches is progressing rapidly. For instance, Qian et al. classified their multigranulation rough sets into two categories: one is the optimistic case and the other is the pessimistic case. Yang et al. [34] generalized the optimistic and pessimistic multigranulation rough sets into fuzzy environment and then proposed the multigranulation fuzzy rough sets models. Furthermore, Qian et al. also proposed a positive approximation [35, 36], which can be used to accelerate a heuristic process of attribute reduction. Since the positive approximation uses a preference ordering, which can make the granulation space finer step by step, that is, a finer granulation space can be obtained by last granulation space, then the positive approximation also reflects the thinking of multigranulation. Bittner and Smith [37, 38] proposed the concept of granular partition, which provides what may be thought of as hierarchical family of partial equivalence relations. Khan and Banerjee [39] studied the rough set approach to multiple-source information system, which reflects the situation where information arrives from multiple sources. Wu and Leung [40, 41] investigated a new knowledge representation system, which is called the multiscale information system. In such system, the data are represented by different scales at different level of granulations, and the granular information is transformed from a finer to a coarser level of granulation.

It must be noticed that the multiscale information system is a very important knowledge representation approach; it can help us to analyze data from the viewpoint of different levels of granulations. For example, maps can be hierarchically organized into different scales, from large to small and vice versa. The smaller the scale, the finer the partition that can be obtained; conversely, the bigger the scale, the coarser the partition that can be obtained. However, what Wu and Leung investigated is complete multiscale information systems. Gore, in his influential book Earth in the Balance [42], notes that “We must acknowledge that we never have complete information. Yet we have to make decisions anyway.” This quote illustrates not only the difficulty of making decisions about environmental issues but also the fact that making such decisions with partial information is ultimately inevitable. Therefore, the investigation of incomplete multiscale information system has become a necessity. Different from the complete multiscale information system, since unknown values are existing in incomplete multiscale information system, then the obtained granulation space at each level of granulation is not necessarily a partition but a covering. To solve such problem, Wu and Leung's approach to multiscale information system has to be reexamined in incomplete multiscale information system. This is what will be discussed in our paper.

In the next section, we first introduce some basic notions related to Pawlak's rough set and multiscale information system. The incomplete multiscale information system and rule induction problem are explored in Section 3. In Section 4, the concept of reducts is introduced into descriptors in incomplete multiscale decision system for the deriving of simplified decision rules. We then conclude the paper with a summary and outlook for further research in Section 5.

## 2. Preliminary Knowledge

### 2.1. Rough Set

Formally, an information system [3] can be considered as a pair *S* = (*U*, AT), in which

- *U* is a nonempty finite set of objects; it is called the universe;
- AT is a nonempty finite set of attributes, such that, ∀*a* ∈ AT, *V*_*a*_ is the domain of attribute *a*.

∀*x* ∈ *U*, let us denote by *a*(*x*) the value that *x* holds on *a* (*a* ∈ AT). For an information system *S*, one then can describe the relationship between objects through their attributes values. With respect to a subset of attributes such that *A*⊆AT, an indiscernibility relation [3] IND(*A*) may be defined as

(1) $$\begin{array}{c} \text{IND}\left(A\right)=\left\{\left(x,y\right)\in U^{2}:a\left(x\right)=a\left(y\right),\forall a\in A\right\}. \end{array}$$

The relation IND(*A*) is reflexive, symmetric, and transitive; then IND(*A*) is an equivalence relation. By the indiscernibility relation IND(*A*), one can derive the lower and upper approximations of an arbitrary subset *X* of *U*. They are defined as

(2) $$\begin{array}{c} \underline{A}\left(X\right)=\left\{x\in U:{\left[x\right]}_{A}\subseteq X\right\},\\ \bar{A}\left(X\right)=\left\{x\in U:{\left[x\right]}_{A}\cap X\neq \emptyset \right\}, \end{array}$$

respectively, where [*x*]_*A*_ = {*y* ∈ *U* : (*x*, *y*) ∈ IND(*A*)} is the *A*-equivalence class containing *x*. The pair $\left[\underline{A}(X),\bar{A}(X)\right]$ is referred to as Pawlak's rough set of *X* with respect to the set of attributes *A*.

### 2.2. Multiscale Information System

A multiscale information system [40] is a tuple *S* = (*U*, AT), where

- *U* = {*x*_1_,…, *x*_*n*_} is a nonempty, finite set of objects called the universe of discourse;
- AT = {*a*_1_,…, *a*_*m*_} is a nonempty, finite set of attributes, and each *a*_*j*_ ∈ *A* is a multiscale attribute; that is, for the same object in *U*, attribute *a*_*j*_ can take on different values at different scales.

In multiscale information system, Wu and Leung [41] assumed that all the attributes have the same number *I* of levels of granulations. Therefore, a multiscale information system can be rewritten as a system such that (*U*, {*a*_*j*_^*k*^ : *k* = 1,2,…, *I*, *j* = 1,2,…, *m*}), where *a*_*j*_^*k*^ : *U* → *V*_*a*_*j*_^*k*^_ is a surjective function and *V*_*a*_*j*_^*k*^_ is the domain of the *k*th scale attribute *a*_*j*_^*k*^. For the set of the *k*th scale attributes AT^*k*^ = {*a*_1_^*k*^, *a*_2_^*k*^,…, *a*_*m*_^*k*^}, one then denotes an equivalence relation such that

(3) $$\begin{array}{c} R_{{\text{AT}}^{k}}=\left\{\left(x,y\right)\in U^{2}:a_{j}^{k}\left(x\right)=a_{j}^{k}\left(y\right),j=1,2,\ldots ,m\right\}. \end{array}$$

The partition induced by *R*_AT^*k*^_ is denoted by *U*/*R*_AT^*k*^_ such that

(4) $$\begin{array}{c} \frac{U}{R_{{\text{AT}}^{k}}}=\left\{{\left[x\right]}_{R_{{\text{AT}}^{k}}}:x\in U\right\}, \end{array}$$

where [*x*]_*R*_AT^*k*^__ is the equivalence class that includes *x* at scale *k*.

It should be noticed that, in multiscale information system, since different scales represent different levels of granulations and then there is a hierarchical structure among *I*-scales, such hierarchical structure can be expressed by the inclusion relation among *m* equivalence relations; that is,

(5) $$\begin{array}{c} R_{{\text{AT}}^{1}}\subseteq R_{{\text{AT}}^{2}}\subseteq \cdots \subseteq R_{{\text{AT}}^{I}}. \end{array}$$

A system *S* = (*U*, AT ∪ {*d*}) = (*U*, {*a*_*j*_^*k*^ : *k* = 1,2,…, *I*, *j* = 1,2,…, *m*}∪{*d*}) is referred to as a multiscale decision system, where (*U*, AT) = (*U*, {*a*_*j*_^*k*^ : *k* = 1,2,…, *I*, *j* = 1,2,…, *m*}) is a multiscale information system and *d* ∉ {*a*_*j*_^*k*^ : *k* = 1,2,…, *I*, *j* = 1,2,…, *m*}, *d* : *U* → *V*_*d*_ is a special attribute called decision. Such multiscale decision system can be decomposed into *I* decision systems *S* ^*k*^ = (*U*, {*a*_*j*_^*k*^ : *j* = 1,2,…, *m*}) = (*U*, AT^*k*^ ∪ {*d*}), *k* = 1,2,…, *I*, with the same decision *d*. Each decomposed decision system *S* ^*k*^ represents information on a special level of granulation, that is, scale.

In [40], Wu and Leung said that the multiscale decision system is referred to as consistent if and only if the decision system under the first (finest) level of scale, that is, *S* ^1^ = (*U*, {*a*_*j*_^1^ : *j* = 1,2,…, *m*}) = (*U*, AT^1^ ∪ {*d*}), is consistent; otherwise it is referred to as inconsistent. Following such work, we will propose a more generalized definition of the concept of consistency in multiscale decision system.

**Definition 1 (see [40]).**

Let *S* = (*U*, AT ∪ {*d*}) = (*U*, {*a*_*j*_^*k*^ : *k* = 1,2,…, *I*, *j* = 1,2,…, *m*}∪{*d*}) be a multiscale decision system; then *S* is referred to as *k*-scale consistent if and only if *k*-scale decision system, that is, *S* ^*k*^ = (*U*, {*a*_*j*_^*k*^ : *j* = 1,2,…, *m*}) = (*U*, AT^*k*^ ∪ {*d*}), is consistent; otherwise *S* is referred to as *k*-scale inconsistent.

By Definition 1, we can see that if *k* = 1, 1-scale consistency is same to what have been proposed by Wu and Leung. In such case, we have *R*_AT^1^_⊆*R*_{*d*}_. Moreover, since *R*_AT^1^_⊆*R*_AT^2^_⊆⋯⊆*R*_AT^*I*^_, then we do not always have *R*_AT^2^_⊆*R*_{*d*}_,…, *R*_AT^*I*^_⊆*R*_{*d*}_; it follows that, in *I*-scale levels of granulations, we need only at least one of the levels of granulations to satisfy the condition of consistent; then this type of the consistent, that is, 1-scale consistent, can be referred to as the optimistic consistent in multiscale decision system.

On the other hand, let us consider the *I*-scale consistent. In such case, we have *R*_AT^*I*^_⊆*R*_{*d*}_. Moreover, since *R*_AT^1^_⊆*R*_AT^2^_⊆⋯⊆*R*_AT^*I*^_, then we also have *R*_AT^2^_⊆*R*_{*d*}_,…, *R*_AT^*I*^_⊆*R*_{*d*}_; it follows that, in *I*-scale levels of granulations, we need all the levels of granulations to satisfy the condition of consistent; then this type of the consistent, that is, *I*-scale consistent, can be referred to as the pessimistic consistent in multiscale decision system.

From discussions above, it is not difficult to observe that the optimistic and pessimistic consistent are all special cases of *k*-scale consistent in multiscale decision system.

**Proposition 2 (see [40]).**

Let *S* = (*U*, *AT* ∪ {*d*}) = (*U*, {*a*_*j*_^*k*^ : *k* = 1,2,…, *I*, *j* = 1,2,…, *m*}∪{*d*}) be a multiscale decision system; if *S* is *k*-scale consistent, then *S* is also *k*′-scale consistent, where *k*′ ∈ {1,2,…, *I*} and *k*′ ≤ *k*.

The above proposition tells us that if a multiscale decision system is consistent in a given scale, then such multiscale decision system is also consistent in the scale, which is smaller than the given scale.

**Remark 3 .**

It should be noticed that the inverse of Proposition 2 does not always hold; that is, if the multiscale decision system is consistent in a smaller scale, then such decision system is not always consistent in a bigger scale.

**Definition 4 (see [40]).**

Let *S* = (*U*, AT ∪ {*d*}) = (*U*, {*a*_*j*_^*k*^ : *k* = 1,2,…, *I*, *j* = 1,2,…, *m*}∪{*d*}) be a multiscale decision system; then ∀*X*⊆*U*, the *k*-scale lower and upper approximations of *X* are denoted by $\underline{{\text{AT}}^{k}}(X)$ and $\bar{{\text{AT}}^{k}}(X)$, respectively, where

(6) ATk_(X)=∪{Y∈URATk:Y⊆X}={x∈U:[x]RATk⊆X};ATk¯(X)=∪{Y∈URATk:Y∩X≠∅}={x∈U:[x]RATk∩X≠∅}.

The pair $\left[\underline{{\text{AT}}^{k}}(X),\bar{{\text{AT}}^{k}}(X)\right]$ is referred to as the *k*-scale rough set of *X* in multiscale decision system. Since the *k*-scale rough set shown in Definition 4 is still based on the equivalence relation, then the properties of Pawlak's rough set still satisfy the *k*-scale rough set. We omit these properties in this paper.

By Definition 4, we can see that, in 1-scale lower approximation, we have [*x*]_*R*_AT^1^__⊆*X*. Since *R*_AT^1^_⊆*R*_AT^2^_⊆⋯⊆*R*_AT^*m*^_, then we do not have that [*x*]_*R*_AT^2^__⊆*X*,…, [*x*]_*R*_AT^*I*^__⊆*X*; it follows that, in *I* levels of granulations, we need only at least one of the levels of granulations to satisfy the inclusion condition between equivalence class and target concept; such explanation is compatible with that in Qian et al.'s optimistic multigranulation lower approximation [31, 33]. Moreover, in 1-scale upper approximation, we have [*x*]_*R*_AT^1^__∩*X* ≠ *∅*. Since *R*_AT^1^_⊆*R*_AT^2^_⊆⋯⊆*R*_AT^*m*^_, then we always have [*x*]_*R*_AT^2^__∩*X* ≠ *∅* ⋯ [*x*]_*R*_AT^*I*^__∩*X* ≠ *∅*; it follows that, in *I* levels of granulations, we need all the levels of granulations to satisfy the intersection condition between equivalence class and target concept; such explanation is also compatible with that in Qian et al.'s optimistic multigranulation upper approximation. From this point of view, 1-scale rough set is also referred to as the optimistic multiscale rough set in multiscale decision system.

On the other hand, let us consider *I*-scale lower approximation; we have [*x*]_*R*_AT^*I*^__⊆*X*. Since *R*_AT^1^_⊆*R*_AT^2^_⊆⋯⊆*R*_AT^*I*^_, then we also have [*x*]_*R*_AT^1^__⊆*X*,…, [*x*]_*R*_AT^*I*−1^__⊆*X*; it follows that, in *I* levels of granulations, we need all the levels of granulations to satisfy the inclusion condition between equivalence class and target concept; such explanation is compatible with that in Qian et al.'s pessimistic multigranulation lower approximation [32]. Moreover, in *I*-scale upper approximation, we have [*x*]_*R*_AT^*I*^__∩*X* ≠ *∅*. Since *R*_AT^1^_⊆*R*_AT^2^_⊆⋯⊆*R*_AT^*m*^_, then we do not always have [*x*]_*R*_AT^1^__∩*X* ≠ *∅* ⋯ [*x*]_*R*_AT^*I*−1^__∩*X* ≠ *∅*; it follows that, in *I* levels of granulations, we need at least one of the levels of granulations to satisfy the intersection condition between equivalence class and target concept; such explanation is also compatible with that in Qian et al.'s pessimistic multigranulation upper approximation. From this point of view, *I*-scale rough set is also referred to as the pessimistic multiscale rough set in multiscale decision system.

**Proposition 5 (see [40]).**

Let *S* = (*U*, *AT* ∪ {*d*}) = (*U*, {*a*_*j*_^*k*^ : *k* = 1,2,…, *I*, *j* = 1,2,…, *m*}∪{*d*}) be a multiscale decision system; then, ∀*X*⊆*U*, one has

(7) $$\begin{array}{c} \underline{{\text{AT}}^{1}}\left(X\right)⊇\underline{{\text{AT}}^{2}}\left(X\right)⊇\cdots ⊇\underline{{\text{AT}}^{I}}\left(X\right);\\ \bar{{\text{AT}}^{1}}\left(X\right)\subseteq \bar{{\text{AT}}^{2}}\left(X\right)\subseteq \cdots \subseteq \bar{{\text{AT}}^{I}}\left(X\right). \end{array}$$

The above proposition tells us that, with the monotonous increasing of levels of granulations, the *k*-scale lower approximations become smaller while the *k*-scale upper approximations become bigger. In other words, we can obtain a string of rough sets through different levels of granulations in multiscale decision system.

The accuracy of *k*-scale rough approximation is defined by

(8) $$\begin{array}{c} \alpha^{k}\left(X\right)=\frac{\left|\underline{{\text{AT}}^{k}}\left(X\right)\right|}{\left|\bar{{\text{AT}}^{k}}\left(X\right)\right|}, \end{array}$$

where |*X*| denotes the cardinal number of set *X*. Obviously, 0 ≤ *α* ^*k*^(*X*) ≤ 1 holds.

**Proposition 6 (see [40]).**

Let *S* = (*U*, *AT* ∪ {*d*}) = (*U*, {*a*_j_^*k*^ : *k* = 1,2,…, *I*, *j* = 1,2,…, *m*}∪{*d*}) be a multiscale decision system; then, ∀*X*⊆*U*, one has

(9) $$\begin{array}{c} \alpha^{1}\left(X\right)\geq \alpha^{2}\left(X\right)\geq \cdots \geq \alpha^{I}\left(X\right). \end{array}$$

## 3. Incomplete Multiscale Information System 

### 3.1. *k*-Scale Descriptors Based Rough Set

An incomplete multiscale information system is still denoted by *S* = (*U*, AT) in this paper. Given an incomplete multiscale information system *S*, if *a*_*j*_^*k*^(*x*) = ∗, then we say that the value of object *x* is unknown on the attribute *a*_*j*_ in terms of the *k*-scale. Moreover, we assume that the unknown value ∗ can be compared with any other values in the domain of the corresponding attributes [4, 5]. Therefore, we use the descriptor based rough set for analyzing the incomplete multiscale information system.

In the discussion to follow, the symbols ∧ and ∨ denote the logical connectives “and” (conjunction) and “or” (disjunction), respectively [15]. Given an incomplete multiscale information system *S*, if *A* ^*k*^⊆AT^*k*^, then any attribute-value pair (*a*_*j*_^*k*^, *v*_*j*_^*k*^) is called an *A* ^*k*^ *-atomic property* where *a*_*j*_^*k*^ ∈ *A* ^*k*^ and *v*_*j*_^*k*^ ∈ *V*_*a*_*j*_^*k*^_. Any *A* ^*k*^-atomic property or conjunction of different *A* ^*k*^-atomic properties is called the *A* ^*k*^ *-descriptor*. If (*a*_*j*_^*k*^, *v*_*j*_^*k*^) is the atomic property occurring in *A* ^*k*^-descriptor *t* ^*k*^, we simply say that (*a*_*j*_^*k*^, *v*_*j*_^*k*^) ∈ *t* ^*k*^. Obviously, *t* ^*k*^ is constructed at scale *k*; it can also be called a *k*-scale descriptor.

Let *t* ^*k*^ be an *A* ^*k*^-descriptor; if, for all (*a*_*j*_^*k*^, *v*_*j*_^*k*^) ∈ *t* ^*k*^, we have (*a*_*j*_^*k*^, *v*_*j*_^*k*^) ∈ *s* ^*k*^, that is, *t* ^*k*^ is constructed from a subset of atomic properties occurring in *s* ^*k*^, then we say *t* ^*k*^ is* coarser* than *s* ^*k*^ or *s* ^*k*^ is* finer* than *t* ^*k*^ and is denoted by *t* ^*k*^⪰*s* ^*k*^ or *s* ^*k*^⪯*t* ^*k*^. If *t* ^*k*^ is constructed from a proper subset of atomic properties occurring in *s* ^*k*^, then we say *t* ^*k*^ is* properly coarser* than *s* ^*k*^ and is denoted by *t* ^*k*^≻*s* ^*k*^ or *s* ^*k*^≺*t* ^*k*^.

Let *t* ^*k*^ be an *A* ^*k*^-descriptor; the attributes set occurring in *t* ^*k*^ is denoted by *A* ^*k*^(*t* ^*k*^). Moreover, if *t* ^*k*^ is an *A* ^*k*^-descriptor and *A* ^*k*^(*t* ^*k*^) = *A* ^*k*^, then *t* ^*k*^ is called* full A* ^*k*^ *-descriptor*. Here, suppose that ∧_*a*_*j*_^*k*^∈*A*^*k*^_(*a*_*j*_^*k*^, *v*_*j*_^*k*^) is a full *A* ^*k*^-descriptor; we denote

(10) ||⋀ajk∈Ak(ajk,vjk)||={x∈U:∀ajk∈Ak,ajk(x)=∗∨ajk(x)=vjk};

then ||∧_*a*_*j*_^*k*^∈*A*^*k*^_(*a*_*j*_^*k*^, *v*_*j*_^*k*^)|| is referred to as the support of ∧_*a*_*j*_^*k*^∈*A*^*k*^_(*a*_*j*_^*k*^, *v*_*j*_^*k*^).

Here, let us denote

(11) $$\begin{array}{c} \text{DES}\left(A^{k}\right)=\left\{t^{k}:t^{k}\;\text{is}\;\text{an}\;A^{k}\text{-descriptor},||t^{k}||\neq \emptyset \right\};\\ \text{FDES}\left(A^{k}\right)=\left\{t^{k}:t^{k}\in \text{DES}\left(A^{k}\right),A^{k}\left(t^{k}\right)=A^{k}\right\}. \end{array}$$

By the descriptor technique, the universe *U* could be partitioned into several subsets that may overlap at scale *k*, and the result is denoted by *U*/*A* ^*k*^ such that

(12) $$\begin{array}{c} \frac{U}{A^{k}}=\left\{||t^{k}||:t^{k}\in \text{FDES}\left(A^{k}\right)\right\}. \end{array}$$

In complete multiscale decision system, the hierarchical structure is represented by a partial relation among different equivalence relations or among different partitions. In incomplete multiscale decision system, since, for each level of granulation, we can obtain a family of the supports of the descriptors, which form coverings on the universe of discourse, and then we can use those supports of the descriptors to represent the hierarchical structure such that

(13) $$\begin{array}{c} \frac{U}{{\text{AT}}^{1}}⊑\frac{U}{{\text{AT}}^{2}}⊑\cdots ⊑\frac{U}{{\text{AT}}^{I}}, \end{array}$$

where, ∀*k*, *k*′ ∈ {1,2,…, *I*} and *k*′ ≤ *k*, *U*/AT^*k*′^⊑*U*/AT^*k*^ means that the following two conditions hold:

1. ∀||*t* ^*k*^|| ∈ *U*/AT^*k*^, there must be ||*t* ^*k*′^|| ∈ *U*/AT^*k*′^ such that ||*t* ^*k*′^||⊆||*t* ^*k*^||;
2. ∀||*t* ^*k*′^|| ∈ *U*/AT^*k*′^, there must be ||*t* ^*k*^|| ∈ *U*/AT^*k*^ such that ||*t* ^*k*′^||⊆||*t* ^*k*^||.

**Remark 7 .**

It should be noticed that if *U*/AT^*k*′^ and *U*/AT^*k*^ are all partitions, then condition (1) implies condition (2) or condition (2) implies condition (1); it follows that only one of the above conditions is needed. However, since, in incomplete multiscale information system, *U*/AT^*k*′^ and *U*/AT^*k*^ may be the coverings instead of the partitions, then the above two conditions are needed simultaneously.

The above two conditions for expressing the hierarchical structure in incomplete multiscale information system are consistent with the basic thinking of surjective function. In other words, in an incomplete multiscale information system *S* = (*U*, AT ∪ {*d*}) = (*U*, {*a*_*j*_^*k*^ : *k* = 1,2,…, *I*, *j* = 1,2,…, *m*}∪{*d*}), ∀*k*, *k*′ ∈ {1,2,…, *I*} and *k*′ ≤ *k*, a surjective function can be defined as

(14) $$\begin{array}{c} g_{k^{\prime }k}:\frac{U}{{\text{AT}}^{k^{\prime }}}⟶\frac{U}{{\text{AT}}^{k}}. \end{array}$$

Such surjective function transforms the granulation spaces from a smaller scale to a bigger scale in the incomplete multiscale information system.

**Example 8 .**

Table 1 shows an example of incomplete multiscale decision system *S* = (*U*, AT ∪ {*d*}) = (*U*, {*a*_*j*_^*k*^ : *k* = 1,2,…, *I*, *j* = 1,2,…, *m*}∪{*d*}), where *U* = {*x*_1_, *x*_2_,…, *x*_20_}, AT = {*a*_1_, *a*_2_, *a*_3_, *a*_4_} is the set of the condition attributes, and *d* is the decision attribute. The system has three levels of granulations, where “*G*,” “*F*,” “*B*,” “*L*,” “*M*,” “*H*,” “*Y*,” and “*N*” stand for, respectively, “good,” “fair,” “bad,” “low,” “medium,” “high,” “yes,” and “no.”

By the descriptor technique we mentioned above, it is not difficult to obtain the descriptors and their supports in each level of granulation. The results of full AT^1^, AT^2^, and AT^3^ descriptors are shown in Tables 2, 3, and 4, respectively.

**Definition 9 .**

Let *S* = (*U*, AT ∪ {*d*}) = (*U*, {*a*_*j*_^*k*^ : *k* = 1,2,…, *I*, *j* = 1,2,…, *m*}∪{*d*}) be an incomplete multiscale decision system; then *S* is referred to as *k*-scale consistent if and only if *k*-scale decision system *S* ^*k*^ = (*U*, {*a*_*j*_^*k*^ : *j* = 1,2,…, *m*}) = (*U*, AT^*k*^ ∪ {*d*}) is consistent; that is, *U*/AT^*k*^⊑*U*/IND({*d*}); otherwise *S* is referred to as *k*-scale inconsistent.

Similar to the complete case, the 1-scale consistent incomplete multiscale decision system is referred to as the optimistic consistent incomplete multiscale decision system while the *I*-scale consistent incomplete multiscale decision system is referred to as the pessimistic consistent incomplete multiscale decision system.

**Proposition 10 .**

Let *S* = (*U*, *AT* ∪ {*d*}) = (*U*, {*a*_*j*_^*k*^ : *k* = 1,2,…, *I*, *j* = 1,2,…, *m*}∪{*d*}) be an incomplete multiscale decision system; if *S* is *k*-scale consistent, then *S* is also *k*′-scale consistent, where *k*′ ∈ {1,2,…, *I*} and *k*′ ≤ *k*.

**Remark 11 .**

It should be noticed that the inverse of Proposition 10 does not always hold; that is, if the incomplete multiscale decision system is consistent in a smaller scale, then such incomplete decision system is not always consistent in a bigger scale.

**Definition 12 .**

Let *S* = (*U*, AT ∪ {*d*}) = (*U*, {*a*_*j*_^*k*^ : *k* = 1,2,…, *I*, *j* = 1,2,…, *m*}∪{*d*}) be an incomplete multiscale decision system; then, ∀*X*⊆*U*, the *k*-scale lower and upper approximations of *X* are denoted by $\underline{{\text{AT}}_{\text{DES}}^{k}}(X)$ and $\bar{{\text{AT}}_{\text{DES}}^{k}}(X)$, respectively, where

(15) $$\begin{array}{c} \underline{{\text{AT}}_{\text{DES}}^{k}}\left(X\right)=\left\{||t^{k}||:t^{k}\in \text{FDES}\left({\text{AT}}^{k}\right),||t^{k}||\subseteq X\right\}; \end{array}$$

(16) $$\begin{array}{c} \bar{{\text{AT}}_{\text{DES}}^{k}}\left(X\right)=\left\{||t^{k}||:t^{k}\in \text{FDES}\left({\text{AT}}^{k}\right),||t^{k}||\cap X\neq \emptyset \right\}. \end{array}$$

By Definition 12, the *k*-scale boundary region of *X* is then

(17) $$\begin{array}{c} \text{B}{\text{N}}_{\text{DES}}^{{\text{AT}}^{k}}\left(X\right)=\bar{{\text{AT}}_{\text{DES}}^{k}}\left(X\right)-\underline{{\text{AT}}_{\text{DES}}^{k}}\left(X\right). \end{array}$$

**Example 13 .**

Take, for instance, Table 1; since the decision attribute partitions the universe into three disjoint subsets such that

(18) $$\begin{array}{c} \frac{U}{\text{IND}\left(\left\{d\right\}\right)}=\left\{X_{1},X_{2},X_{3}\right\}, \end{array}$$

where *X*_1_ = {*x*_1_, *x*_2_, *x*_3_, *x*_13_, *x*_14_, *x*_15_}, *X*_2_ = {*x*_4_, *x*_5_, *x*_6_, *x*_7_, *x*_8_, *x*_9_, *x*_10_, *x*_11_, *x*_12_}, *X*_3_ = {*x*_16_, *x*_17_, *x*_18_, *x*_19_, *x*_20_}, then by Definition 12, we obtain the following 1-scale, 2-scale, and 3-scale rough sets:

(1)* 1-scale lower and upper approximations:*
$\underline{\text{A}{\text{T}}_{\text{DES}}^{1}}\left(X_{1}\right)=\left\{||t_{3}^{1}||,||t_{8}^{1}||,||t_{10}^{1}||,||t_{15}^{1}||,||t_{23}^{1}||,||t_{26}^{1}||\right\}$,
$\bar{\text{A}{\text{T}}_{\text{DES}}^{1}}\left(X_{1}\right)=\{||t_{1}^{1}||$, ||*t*_3_^1^||, ||*t*_5_^1^||, ||*t*_7_^1^||,||*t*_8_^1^||, ||*t*_9_^1^||, ||*t*_10_^1^||, ||*t*_14_^1^||, ||*t*_15_^1^||, ||*t*_19_^1^||, ||*t*_23_^1^||, ||*t*_26_^1^||},
$\underline{\text{A}{\text{T}}_{\text{DES}}^{1}}(X_{2})=\{||t_{2}^{1}||$, ||*t*_4_^1^||, ||*t*_6_^1^||, ||*t*_11_^1^||, ||*t*_12_^1^||,||*t*_13_^1^||, ||*t*_16_^1^||, ||*t*_17_^1^||, ||*t*_18_^1^||, ||*t*_20_^1^||, ||*t*_21_^1^||, ||*t*_22_^1^||, ||*t*_25_^1^||},
$\bar{\text{A}{\text{T}}_{\text{DES}}^{1}}(X_{2})=\{||t_{2}^{1}||$, ||*t*_4_^1^||, ||*t*_6_^1^||, ||*t*_9_^1^||, ||*t*_11_^1^||, ||*t*_12_^1^||, ||*t*_13_^1^||, ||*t*_14_^1^||, ||*t*_16_^1^||, ||*t*_17_^1^||, ||*t*_18_^1^||, ||*t*_19_^1^||, ||*t*_20_^1^||, ||*t*_21_^1^||, ||*t*_22_^1^||, ||*t*_25_^1^||},
$\underline{\text{A}{\text{T}}_{\text{DES}}^{1}}(X_{3})=\{||t_{24}^{1}||,||t_{27}^{1}||\}$,
$\bar{\text{A}{\text{T}}_{\text{DES}}^{1}}(X_{3})=\{||t_{1}^{1}||,||t_{5}^{1}||||||,||t_{7}^{1}||,||t_{24}^{1}||,||t_{27}^{1}||\}$;
(2)* 2-scale lower and upper approximations:*
$\underline{{\text{AT}}_{\text{DES}}^{2}}(X_{1})=\{||t_{7}^{2}||,||t_{8}^{2}||\}$,
$\bar{{\text{AT}}_{\text{DES}}^{2}}(X_{1})=\{||t_{1}^{2}||,||t_{3}^{2}||,||t_{7}^{2}||,||t_{8}^{2}||\}$,
$\underline{{\text{AT}}_{\text{DES}}^{2}}(X_{2})=\{||t_{2}^{2}||,||t_{4}^{2}||,||t_{5}^{2}||,||t_{6}^{2}||\}$,
$\bar{{\text{AT}}_{\text{DES}}^{2}}(X_{2})=\{||t_{2}^{2}||,||t_{3}^{2}||,||t_{4}^{2}||,||t_{5}^{2}||,||t_{6}^{2}||\}$,
$\underline{{\text{AT}}_{\text{DES}}^{2}}(X_{3})=\{||t_{9}^{2}||,||t_{10}^{2}||,||t_{11}^{2}||\}$,
$\bar{{\text{AT}}_{\text{DES}}^{2}}(X_{3})=\{||t_{1}^{2}||,||t_{9}^{2}||,||t_{10}^{2}||,||t_{11}^{2}||\}$;
(3)* 3-scale lower and upper approximations:*
$\underline{{\text{AT}}_{\text{DES}}^{3}}(X_{1})=\emptyset$,
$\bar{{\text{AT}}_{\text{DES}}^{3}}(X_{1})=\{||t_{1}^{3}||,||t_{2}^{3}||\}$,
$\underline{{\text{AT}}_{\text{DES}}^{3}}(X_{2})=\{||t_{4}^{3}||\}$,
$\bar{{\text{AT}}_{\text{DES}}^{3}}(X_{2})=\{||t_{2}^{3}||,||t_{3}^{3}||,||t_{4}^{3}||\}$,
$\underline{{\text{AT}}_{\text{DES}}^{3}}(X_{3})=\emptyset$,
$\bar{{\text{AT}}_{\text{DES}}^{3}}(X_{3})=\{||t_{1}^{3}||,||t_{3}^{3}||\}$.

By the above computations, we can see that Table 1 is inconsistent at each level of granulation, that is, each scale.

**Proposition 14 .**

Let *S* = (*U*, AT ∪ {*d*}) = (*U*, {*a*_*j*_^*k*^ : *k* = 1,2,…, *I*, *j* = 1,2,…, *m*}∪{*d*}) be an incomplete multiscale decision system; then, ∀*X*⊆*U*, one has

(19) $$\begin{array}{c} \cup \underline{{AT}_{DES}^{1}}\left(X\right)⊇\cup \underline{{AT}_{DES}^{2}}\left(X\right)⊇\cdots ⊇\cup \underline{{AT}_{DES}^{I}}\left(X\right);\\ \cup \bar{{AT}_{DES}^{1}}\left(X\right)\subseteq \cup \bar{{AT}_{DES}^{2}}\left(X\right)\subseteq \cdots \subseteq \cup \bar{{AT}_{DES}^{I}}\left(X\right);\\ \cup {BN}_{DES}^{{AT}^{1}}\left(X\right)\subseteq \cup {BN}_{DES}^{{AT}^{2}}\left(X\right)\subseteq \cdots \subseteq \cup {BN}_{DES}^{{AT}^{I}}\left(X\right). \end{array}$$

The results in Proposition 14 are consistent with those in Proposition 5; that is, with the variety of levels of granulations in incomplete multiscale decision system, the lower approximations, upper approximations, and boundary regions are monotonic.

### 3.2. *k*-Scale Decision Rules

The end result of rough set is a representation of the information contained in the data system considered in terms of “if… then…” decision rules [43, 44]. Since an incomplete multiscale decision system contains a family of the systems with different levels of granulations, then, given an incomplete multiscale decision system, one can derive decision rules at each scale. For example, suppose that *S* = (*U*, AT ∪ {*d*}) = (*U*, {*a*_*j*_^*k*^ : *k* = 1,2,…, *I*, *j* = 1,2,…, *m*}∪{*d*}) be an incomplete multiscale decision system; then a *k*-scale decision rule is represented by

(20) $$\begin{array}{c} r^{k}:t^{k}⟶s, \end{array}$$

where *t* ^*k*^ ∈ FDES(AT^*k*^), *s* = (*d*, *w*), *w* ∈ *V*_*d*_, and *t* ^*k*^ and *s* are, respectively, called the condition and decision parts of the rule *r* ^*k*^.

For each *k*-scale decision rule *r* ^*k*^ : *t* ^*k*^ → *s*, we associate a quantitative measure, called the certainty, of *r* ^*k*^ and it is defined by

(21) $$\begin{array}{c} \text{Cer}\left(r^{k}\right)=\text{Cer}\left(t^{k}⟶s\right)=\frac{\text{Card}\left(||t^{k}||\cap ||s||\right)}{\text{Card}\left(||t^{k}||\right)}. \end{array}$$

A *k*-scale decision rule *r* ^*k*^ : *t* ^*k*^ → *s* is referred to as certain if and only if Cer(*r* ^*k*^) = 1; a *k*-scale decision rule *r* ^*k*^ : *t* ^*k*^ → *s* is referred to as possible if and only if 0 < Cer(*r* ^*k*^) < 1. Similar to the traditional rough set approach, the certain decision rules are supported by the descriptors in lower approximation while the possible rules are supported by the descriptors in boundary region. In other words, if $||t^{k}||\in \underline{{\text{AT}}_{\text{DES}}^{k}}(||\left(d,w\right)||)$, then *t* ^*k*^ → *s* is a certain decision rule; if ||*t* ^*k*^|| ∈ BN_DES_^AT^*k*^^(||(*d*, *w*)||), then *t* ^*k*^ → *s* is a possible decision rule.

In an incomplete multiscale decision system *S* = (*U*, AT ∪{*d*}) = (*U*, {*a*_*j*_^*k*^ : *k* = 1,2,…, *I*, *j* = 1,2,…, *m*}∪{*d*}),

1. since *U*/AT^1^⊑*U*/AT^2^⊑⋯⊑*U*/AT^*I*^, then, ∀||*t* ^*k*^|| ∈ *U*/AT^*k*^, there must be ||*t* ^*k*′^|| ∈ *U*/AT^*k*′^ such that ||*t* ^*k*′^||⊆||*t* ^*k*^||; it tells us that if we have a certain *k*-scale decision rule such that *r* ^*k*^ : *t* ^*k*^ → *s*, then we can also obtain a certain *k*′-scale decision rule; that is, *r* ^*k*′^ : *t* ^*k*′^ → *s*;
2. since *U*/AT^1^⊑*U*/AT^2^⊑⋯⊑*U*/AT^*I*^, then, ∀||*t* ^*k*′^|| ∈ *U*/AT^*k*′^, there must be ||*t* ^*k*^|| ∈ *U*/AT^*k*^ such that ||*t* ^*k*′^||⊆||*t* ^*k*^||; it tells us that if we have a possible *k*′-scale decision rule such that *r* ^*k*′^ : *t* ^*k*′^ → *s*, then we can also obtain a possible *k*-scale decision rule; that is, *r* ^*k*^ : *t* ^*k*^ → *s*.

**Example 15 .**

Following the results of approximations we obtained in Example 13, it is not difficult to derive the following decision rules at 3 different levels of granulations in Table 1:

(1)* 1-scale decision rules:*
  (a) * 1-scale certain decision rules:*
*r*_1_^1^ : *a*_1_^1^(*x*) = 1∧*a*_2_^1^(*x*) = 3∧*a*_3_^1^(*x*) = 5∧*a*_4_^1^(*x*) = 2 → *d*(*x*) = 1 // supported by *t*_3_^1^,
*r*_2_^1^ : *a*_1_^1^(*x*) = 3∧*a*_2_^1^(*x*) = 3∧*a*_3_^1^(*x*) = 2∧*a*_4_^1^(*x*) = 2 → *d*(*x*) = 1 // supported by *t*_8_^1^,
*r*_3_^1^ : *a*_1_^1^(*x*) = 4∧*a*_2_^1^(*x*) = 3∧*a*_3_^1^(*x*) = 5∧*a*_4_^1^(*x*) = 2 → *d*(*x*) = 1 // supported by *t*_10_^1^,
*r*_4_^1^ : *a*_1_^1^(*x*) = 5∧*a*_2_^1^(*x*) = 3∧*a*_3_^1^(*x*) = 5∧*a*_4_^1^(*x*) = 2 → *d*(*x*) = 1 // supported by *t*_15_^1^,
*r*_5_^1^ : *a*_1_^1^(*x*) = 7∧*a*_2_^1^(*x*) = 3∧*a*_3_^1^(*x*) = 5∧*a*_4_^1^(*x*) = 2 → *d*(*x*) = 1 // supported by *t*_23_^1^,
*r*_6_^1^ : *a*_1_^1^(*x*) = 8∧*a*_2_^1^(*x*) = 3∧*a*_3_^1^(*x*) = 5∧*a*_4_^1^(*x*) = 2 → *d*(*x*) = 1 // supported by *t*_26_^1^,
*r*_7_^1^ : *a*_1_^1^(*x*) = 1∧*a*_2_^1^(*x*) = 1∧*a*_3_^1^(*x*) = 3∧*a*_4_^1^(*x*) = 1 → *d*(*x*) = 2 // supported by *t*_2_^1^,
*r*_8_^1^ : *a*_1_^1^(*x*) = 2∧*a*_2_^1^(*x*) = 1∧*a*_3_^1^(*x*) = 3∧*a*_4_^1^(*x*) = 1 → *d*(*x*) = 2 // supported by *t*_4_^1^,
*r*_9_^1^ : *a*_1_^1^(*x*) = 3∧*a*_2_^1^(*x*) = 1∧*a*_3_^1^(*x*) = 3∧*a*_4_^1^(*x*) = 1 → *d*(*x*) = 2 // supported by *t*_6_^1^,
*r*_10_^1^ : *a*_1_^1^(*x*) = 4∧*a*_2_^1^(*x*) = 4∧*a*_3_^1^(*x*) = 1∧*a*_4_^1^(*x*) = 1 → *d*(*x*) = 2 // supported by *t*_11_^1^,
*r*_11_^1^ : *a*_1_^1^(*x*) = 4∧*a*_2_^1^(*x*) = 4∧*a*_3_^1^(*x*) = 3∧*a*_4_^1^(*x*) = 3 → *d*(*x*) = 2 // supported by *t*_12_^1^
*r*_12_^1^ : *a*_1_^1^(*x*) = 5∧*a*_2_^1^(*x*) = 1∧*a*_3_^1^(*x*) = 3∧*a*_4_^1^(*x*) = 1 → *d*(*x*) = 2 // supported by *t*_13_^1^,
*r*_13_^1^ : *a*_1_^1^(*x*) = 5∧*a*_2_^1^(*x*) = 5∧*a*_3_^1^(*x*) = 4∧*a*_4_^1^(*x*) = 1 → *d*(*x*) = 2 // supported by *t*_16_^1^,
*r*_14_^1^ : *a*_1_^1^(*x*) = 5∧*a*_2_^1^(*x*) = 5∧*a*_3_^1^(*x*) = 4∧*a*_4_^1^(*x*) = 4 → *d*(*x*) = 2 // supported by *t*_17_^1^,
*r*_15_^1^ : *a*_1_^1^(*x*) = 6∧*a*_2_^1^(*x*) = 1∧*a*_3_^1^(*x*) = 3∧*a*_4_^1^(*x*) = 1 → *d*(*x*) = 2 // supported by *t*_18_^1^,
*r*_16_^1^ : *a*_1_^1^(*x*) = 6∧*a*_2_^1^(*x*) = 6∧*a*_3_^1^(*x*) = 5∧*a*_4_^1^(*x*) = 4 → *d*(*x*) = 2 // supported by *t*_20_^1^,
*r*_17_^1^ : *a*_1_^1^(*x*) = 6∧*a*_2_^1^(*x*) = 6∧*a*_3_^1^(*x*) = 5∧*a*_4_^1^(*x*) = 2 → *d*(*x*) = 2 // supported by *t*_21_^1^,
*r*_18_^1^ : *a*_1_^1^(*x*) = 7∧*a*_2_^1^(*x*) = 1∧*a*_3_^1^(*x*) = 3∧*a*_4_^1^(*x*) = 1 → *d*(*x*) = 2 // supported by *t*_22_^1^,
*r*_19_^1^ : *a*_1_^1^(*x*) = 8∧*a*_2_^1^(*x*) = 1∧*a*_3_^1^(*x*) = 3∧*a*_4_^1^(*x*) = 1 → *d*(*x*) = 2 // supported by *t*_25_^1^,
*r*_20_^1^ : *a*_1_^1^(*x*) = 7∧*a*_2_^1^(*x*) = 7∧*a*_3_^1^(*x*) = 6∧*a*_4_^1^(*x*) = 5 → *d*(*x*) = 3 // supported by *t*_24_^1^,
*r*_21_^1^ : *a*_1_^1^(*x*) = 8∧*a*_2_^1^(*x*) = 7∧*a*_3_^1^(*x*) = 6∧*a*_4_^1^(*x*) = 6 → *d*(*x*) = 3 // supported by *t*_27_^1^;
  (b) * 1-scale possible decision rules:*
*r*_1_^′1^ : *a*_1_^1^(*x*) = 1∧*a*_2_^1^(*x*) = 1∧*a*_3_^1^(*x*) = 1∧*a*_4_^1^(*x*) = 1 → *d*(*x*) = 1, Cer (*r*_1_^′1^) = 0.5 // supported by *t*_1_^1^,
*r*_2_^′1^ : *a*_1_^1^(*x*) = 2∧*a*_2_^1^(*x*) = 2∧*a*_3_^1^(*x*) = 1∧*a*_4_^1^(*x*) = 1 → *d*(*x*) = 1, Cer (*r*_2_^′1^) = 0.5 // supported by *t*_5_^1^,
*r*_3_^′1^ : *a*_1_^1^(*x*) = 3∧*a*_2_^1^(*x*) = 3∧*a*_3_^1^(*x*) = 2∧*a*_4_^1^(*x*) = 2 → *d*(*x*) = 1, Cer (*r*_3_^′1^) = 0.5 // supported by *t*_7_^1^,
*r*_4_^′1^ : *a*_1_^1^(*x*) = 4∧*a*_2_^1^(*x*) = 1∧*a*_3_^1^(*x*) = 3∧*a*_4_^1^(*x*) = 1 → *d*(*x*) = 1, Cer (*r*_4_^′1^) = 0.5 // supported by *t*_9_^1^,
*r*_5_^′1^ : *a*_1_^1^(*x*) = 5∧*a*_2_^1^(*x*) = 2∧*a*_3_^1^(*x*) = 4∧*a*_4_^1^(*x*) = 1 → *d*(*x*) = 1, Cer (*r*_5_^′1^) = 0.5 // supported by *t*_14_^1^,
*r*_6_^′1^ : *a*_1_^1^(*x*) = 6∧*a*_2_^1^(*x*) = 3∧*a*_3_^1^(*x*) = 5∧*a*_4_^1^(*x*) = 2 → *d*(*x*) = 1, Cer (*r*_6_^′1^) = 0.5 // supported by *t*_19_^1^,
*r*_7_^′1^ : *a*_1_^1^(*x*) = 4∧*a*_2_^1^(*x*) = 1∧*a*_3_^1^(*x*) = 3∧*a*_4_^1^(*x*) = 1 → *d*(*x*) = 2, Cer (*r*_7_^′1^) = 0.5 // supported by *t*_9_^1^,
*r*_8_^′1^ : *a*_1_^1^(*x*) = 5∧*a*_2_^1^(*x*) = 2∧*a*_3_^1^(*x*) = 4∧*a*_4_^1^(*x*) = 1 → *d*(*x*) = 2, Cer (*r*_8_^′1^) = 0.5 // supported by *t*_14_^1^,
*r*_9_^′1^ : *a*_1_^1^(*x*) = 6∧*a*_2_^1^(*x*) = 3∧*a*_3_^1^(*x*) = 5∧*a*_4_^1^(*x*) = 2 → *d*(*x*) = 2, Cer (*r*_9_^′1^) = 0.5 // supported by *t*_19_^1^,
*r*_10_^′1^ : *a*_1_^1^(*x*) = 1∧*a*_2_^1^(*x*) = 1∧*a*_3_^1^(*x*) = 1∧*a*_4_^1^(*x*) = 1 → *d*(*x*) = 3, Cer (*r*_10_^′1^) = 0.5 // supported by *t*_1_^1^,
*r*_11_^′1^ : *a*_1_^1^(*x*) = 2∧*a*_2_^1^(*x*) = 2∧*a*_3_^1^(*x*) = 1∧*a*_4_^1^(*x*) = 1 → *d*(*x*) = 3, Cer (*r*_11_^′1^) = 0.5 // supported by *t*_5_^1^,
*r*_12_^′1^ : *a*_1_^1^(*x*) = 3∧*a*_2_^1^(*x*) = 3∧*a*_3_^1^(*x*) = 2∧*a*_4_^1^(*x*) = 2 → *d*(*x*) = 1, Cer (*r*_12_^′1^) = 0.5 // supported by *t*_7_^1^;
(2)* 2-scale decision rules:*
  (a) * 2-scale certain decision rules:*
*r*_1_^2^ : *a*_1_^2^(*x*) = *F*∧*a*_2_^2^(*x*) = *G*∧*a*_3_^2^(*x*) = *M*∧*a*_4_^2^(*x*) = *L* → *d*(*x*) = 1 // supported by *t*_7_^2^,
*r*_2_^2^ : *a*_1_^2^(*x*) = *F*∧*a*_2_^2^(*x*) = *G*∧*a*_3_^2^(*x*) = *L*∧*a*_4_^2^(*x*) = *L* → *d*(*x*) = 1 // supported by *t*_8_^2^,
*r*_3_^2^ : *a*_1_^2^(*x*) = *F*∧*a*_2_^2^(*x*) = *G*∧*a*_3_^2^(*x*) = *H*∧*a*_4_^2^(*x*) = *L* → *d*(*x*) = 1 // supported by *t*_9_^2^,
*r*_4_^2^ : *a*_1_^2^(*x*) = *F*∧*a*_2_^2^(*x*) = *G*∧*a*_3_^2^(*x*) = *M*∧*a*_4_^2^(*x*) = *M* → *d*(*x*) = 2 // supported by *t*_2_^2^,
*r*_5_^2^ : *a*_1_^2^(*x*) = *F*∧*a*_2_^2^(*x*) = *F*∧*a*_3_^2^(*x*) = *M*∧*a*_4_^2^(*x*) = *M* → *d*(*x*) = 2 // supported by *t*_4_^2^,
*r*_6_^2^ : *a*_1_^2^(*x*) = *F*∧*a*_2_^2^(*x*) = *F*∧*a*_3_^2^(*x*) = *M*∧*a*_4_^2^(*x*) = *L* → *d*(*x*) = 2 // supported by *t*_5_^2^,
*r*_7_^2^ : *a*_1_^2^(*x*) = *F*∧*a*_2_^2^(*x*) = *B*∧*a*_3_^2^(*x*) = *M*∧*a*_4_^2^(*x*) = *M* → *d*(*x*) = 2 // supported by *t*_6_^2^,
*r*_8_^2^ : *a*_1_^2^(*x*) = *B*∧*a*_2_^2^(*x*) = *B*∧*a*_3_^2^(*x*) = *H*∧*a*_4_^2^(*x*) = *H* → *d*(*x*) = 3 // supported by *t*_10_^2^,
*r*_9_^2^ : *a*_1_^2^(*x*) = *B*∧*a*_2_^2^(*x*) = *F*∧*a*_3_^2^(*x*) = *H*∧*a*_4_^2^(*x*) = *H* → *d*(*x*) = 3 // supported by *t*_11_^2^,
*r*_10_^2^ : *a*_1_^2^(*x*) = *B*∧*a*_2_^2^(*x*) = *G*∧*a*_3_^2^(*x*) = *H*∧*a*_4_^2^(*x*) = *H* → *d*(*x*) = 3 // supported by *t*_12_^2^;
  (b) * 2-scale possible decision rules:*
*r*_1_^′2^ : *a*_1_^2^(*x*) = *G*∧*a*_2_^2^(*x*) = *G*∧*a*_3_^2^(*x*) = *L*∧*a*_4_^2^(*x*) = *L* → *d*(*x*) = 1, Cer (*r*_1_^′2^) = 0.5 // supported by *t*_1_^2^,
*r*_2_^′2^ : *a*_1_^2^(*x*) = *F*∧*a*_2_^2^(*x*) = *G*∧*a*_3_^2^(*x*) = *M*∧*a*_4_^2^(*x*) = *L* → *d*(*x*) = 1, Cer (*r*_2_^′2^) = 0.5 // supported by *t*_3_^2^,
*r*_3_^′2^ : *a*_1_^2^(*x*) = *F*∧*a*_2_^2^(*x*) = *G*∧*a*_3_^2^(*x*) = *M*∧*a*_4_^2^(*x*) = *L* → *d*(*x*) = 2, Cer (*r*_3_^′2^) = 0.5 // supported by *t*_3_^2^,
*r*_4_^′2^ : *a*_1_^2^(*x*) = *G*∧*a*_2_^2^(*x*) = *G*∧*a*_3_^2^(*x*) = *L*∧*a*_4_^2^(*x*) = *L* → *d*(*x*) = 3, Cer (*r*_4_^′2^) = 0.5 // supported by *t*_1_^2^;
(3)* 3-scale decision rules:*
  (a) * 3-scale certain decision rules:*
*r*_1_^3^ : *a*_1_^3^(*x*) = *N*∧*a*_2_^3^(*x*) = *N*∧*a*_3_^3^(*x*) = *N*∧*a*_4_^3^(*x*) = *Y* → *d*(*x*) = 2 // supported by *t*_4_^3^;
  (b) * 3-scale possible decision rules:*
*r*_1_^′3^ : *a*_1_^3^(*x*) = *Y*∧*a*_2_^3^(*x*) = *Y*∧*a*_3_^3^(*x*) = *Y*∧*a*_4_^3^(*x*) = *Y* → *d*(*x*) = 1, Cer (*r*_1_^′3^) = 0.5 // supported by *t*_1_^3^,
*r*_2_^′3^ : *a*_1_^3^(*x*) = *N*∧*a*_2_^3^(*x*) = *Y*∧*a*_3_^3^(*x*) = *N*∧*a*_4_^3^(*x*) = *Y* → *d*(*x*) = 1, Cer (*r*_2_^′3^) = 0.5 // supported by *t*_2_^3^,
*r*_3_^′3^ : *a*_1_^3^(*x*) = *N*∧*a*_2_^3^(*x*) = *Y*∧*a*_3_^3^(*x*) = *N*∧*a*_4_^3^(*x*) = *Y* → *d*(*x*) = 2, Cer (*r*_3_^′3^) = 0.5 // supported by *t*_2_^3^,
*r*_4_^′3^ : *a*_1_^3^(*x*) = *N*∧*a*_2_^3^(*x*) = *N*∧*a*_3_^3^(*x*) = *N*∧*a*_4_^3^(*x*) = *N* → *d*(*x*) = 2, Cer (*r*_4_^′3^) = 0.667 // supported by *t*_3_^3^,
*r*_5_^′3^ : *a*_1_^3^(*x*) = *Y*∧*a*_2_^3^(*x*) = *Y*∧*a*_3_^3^(*x*) = *Y*∧*a*_4_^3^(*x*) = *Y* → *d*(*x*) = 3, Cer (*r*_5_^′3^) = 0.5 // supported by *t*_1_^3^,
*r*_6_^′3^ : *a*_1_^3^(*x*) = *N*∧*a*_2_^3^(*x*) = *N*∧*a*_3_^3^(*x*) = *N*∧*a*_4_^3^(*x*) = *N* → *d*(*x*) = 3, Cer (*r*_6_^′3^) = 0.333 // supported by *t*_3_^3^.

## 4. Reductions in Incomplete Multiscale Decision System 

### 4.1. Reducts of Descriptors

**Definition 16 .**

Let *S* = (*U*, AT ∪ {*d*}) = (*U*, {*a*_*j*_^*k*^ : *k* = 1,2,…, *I*, *j* = 1,2,…, *m*}∪{*d*}) be an incomplete multiscale decision system, *t* ^*k*^ ∈ FDES(AT^*k*^) is a full AT^*k*^-descriptor, *t* ^′*k*^ ∈ DES(AT^*k*^) is an AT^*k*^-descriptor, and *t* ^′*k*^⪰*t* ^*k*^; then *t* ^′*k*^ is referred to as the reduct descriptor of *t* ^*k*^ if and only if the following two conditions hold:

1. ||*t* ^′*k*^|| = ||*t* ^*k*^||;
2. ||*t* ^′′*k*^|| ≠ ||*t* ^*k*^|| for each *t* ^′′*k*^≻*t* ^′*k*^.

By Definition 16, we can see that a reduct descriptor of *t* ^*k*^ is a conjunction of the atomic properties in *t* ^*k*^, which preserves the support of *t* ^*k*^. The reduct descriptor allows us to classify objects with the smallest number of required atomic properties.

To compute the reduct descriptor of *t* ^*k*^, let us define

(22) DIS(tk,y)={{ajk∈ATk:y∉||(ajk,vjk)||,(ajk,vjk)∈tk}:y∉||tk||,∅:otherwise;

then

(23) $$\begin{array}{c} M^{t^{k}}=\left\{\text{DIS}\left(t^{k},y\right):y\notin ||t^{k}||\right\} \end{array}$$

is referred to as the *t* ^*k*^ discernibility matrix.

**Theorem 17 .**

Let *S* = (*U*, *AT* ∪ {*d*}) = (*U*, {*a*_*j*_^*k*^ : *k* = 1,2,…, *I*, *j* = 1,2,…, *m*}∪{*d*}) be an incomplete multiscale decision system in which *t* ^*k*^ ∈ *FDES*(*AT* ^*k*^), *t* ^′*k*^ ∈ *DES*(*AT* ^*k*^), and *t* ^′*k*^⪰*t* ^*k*^; then

 ||*t* ^*k*^|| = ||*t* ^′*k*^||⇔AT(*t* ^′*k*^)∩*DIS*(*t* ^*k*^, *y*) ≠ *∅* for each *DIS*(*t* ^*k*^, *y*) ∈ **M** ^*t*^*k*^^.

**Proof**

⇒: Suppose ∃DIS(*t* ^*k*^, *y*) ∈ **M** ^*t*^*k*^^ such that AT(*t* ^′*k*^)∩DIS(*t* ^*k*^, *y*) = *∅*; then by the definition of DIS(*t* ^*k*^, *y*), we have *y* ∈ ||*t* ^′*k*^|| because *t* ^′*k*^⪰*t* ^*k*^. Moreover, since ||*t* ^′*k*^|| = ||*t* ^*k*^||, then *y* ∈ ||*t* ^*k*^||; such result is contradictive to the condition DIS(*t* ^*k*^, *y*) ∈ **M** ^*t*^*k*^^⇒*y* ∉ ||*t* ^*k*^||.

⇐: Since *t* ^′*k*^⪰*t* ^*k*^, then ||*t* ^′*k*^||⊇||*t* ^*k*^|| holds obviously. Therefore, it must be proved that ||*t* ^′*k*^||⊆||*t* ^*k*^||. For each *y* ∉ ||*t* ^*k*^||, DIS(*t* ^*k*^, *y*) ∈ **M** ^*t*^*k*^^, since AT(*t* ^′*k*^)∩DIS(*t* ^*k*^, *y*) ≠ *∅*; then there must be *a*_*j*_^*k*^ ∈ AT^*k*^(*t* ^′*k*^) such that *y* ∉ ||(*a*_*j*_^*k*^, *v*_*j*_^*k*^)||; it follows that *y* ∉ ||*t* ^′*k*^||, from which we can conclude that *y* ∉ ||*t* ^*k*^||⇒*y* ∉ ||*t* ^′*k*^||; that is, ||*t* ^′*k*^||⊆||*t* ^*k*^||. That completes the proof.

**Definition 18 .**

Let *S* = (*U*, AT ∪ {*d*}) = (*U*, {*a*_*j*_^*k*^ : *k* = 1,2,…, *I*, *j* = 1,2,…, *m*}∪{*d*}) be an incomplete multiscale decision system in which *t* ^*k*^ ∈ FDES(AT^*k*^); then the *t* ^*k*^ discernibility function is defined as

(24) $$\begin{array}{c} \Delta^{t^{k}}=\overset{}{\underset{\text{DIS}(t^{k},y)\in M^{t^{k}}}{⋀}}\overset{}{\underset{}{⋁}}\text{DIS}\left(t^{k},y\right). \end{array}$$

**Theorem 19 .**

Let *S* = (*U*, *AT* ∪ {*d*}) = (*U*, {*a*_*j*_^*k*^ : *k* = 1,2,…, *I*, *j* = 1,2,…, *m*}∪{*d*}) be a multiscale decision system in which *t* ^*k*^ ∈ *FDES*(*AT* ^*k*^), *t* ^′*k*^ ∈ *DES*(*AT* ^*k*^), and *t* ^′*k*^⪰*t* ^*k*^; then

 
*t* ^′*k*^ is a reduct descriptor of *t* ^*k*^ if and only if *AT* ^*k*^(*t* ^′*k*^) is the prime implicant of the *t* ^*k*^ discernibility function Δ^*t*^*k*^^.

**Proof**

⇒: By Theorem 17, we have AT(*t* ^′*k*^)∩DIS(*t* ^*k*^, *y*) ≠ *∅* for each DIS(*t* ^*k*^, *y*) ∈ **M** ^*t*^*k*^^. We claim that, for each *a*_*j*_^*k*^ ∈ AT^*k*^(*t* ^′*k*^), there must be DIS(*t* ^*k*^, *y*) ∈ **M** ^*t*^*k*^^ such that AT(*t* ^′*k*^)∩DIS(*t* ^*k*^, *y*) = {*a*_*j*_^*k*^}. If fact, if ∀DIS(*t* ^*k*^, *y*) ∈ **M** ^*t*^*k*^^, there exist *a*_*j*_^*k*^ ∈ DIS(*t* ^*k*^, *y*) such that |AT(*t* ^′*k*^)∩DIS(*t* ^*k*^, *y*)| > 2, where *a*_*j*_^*k*^ ∈ AT^*k*^(*t* ^′*k*^)∩DIS(*t* ^*k*^, *y*); let *t* ^′′*k*^≻*t* ^′*k*^, where AT^*k*^(*t* ^′′*k*^) = AT^*k*^(*t* ^′*k*^)−{*a*_*j*_^*k*^}; then by Theorem 17 we know ||*t* ^′′*k*^|| = ||*t* ^*k*^||, which contradicts the fact that *t* ^′*k*^ is a reduct descriptor of *t* ^*k*^; it follows that AT^*k*^(*t* ^′*k*^) is the prime implicant of the Δ^*t*^*k*^^.

⇐: If AT^*k*^(*t* ^′*k*^) is the prime implicant of the Δ^*t*^*k*^^, then by Definition 18, we have AT^*k*^(*t*′)∩DIS(*t* ^*k*^, *y*) ≠ *∅* for each DIS(*t* ^*k*^, *y*) ∈ **M** ^*t*^*k*^^. Moreover, for each *a*_*j*_^*k*^ ∈ AT^*k*^(*t* ^′*k*^), there exists DIS(*t* ^*k*^, *y*) ∈ **M** ^*t*^*k*^^ such that AT^*k*^(*t* ^′*k*^)∩DIS(*t* ^*k*^, *y*) = {*a*_*j*_^*k*^}. Consequently, ∀*t* ^′′*k*^≻*t* ^′*k*^ and AT^*k*^(*t* ^′′*k*^) = AT^*k*^(*t* ^′*k*^)−{*a*_*j*_^*k*^}, ||*t* ^′′*k*^|| ≠ ||*t* ^*k*^||; we then conclude that *t* ^′*k*^ is a reduct descriptor of *t* ^*k*^. That completes the proof.

### 4.2. Lower Approximation and Boundary Region Reduct Descriptors

In Section 3.2, we have mentioned that the certain decision rules can be generated from the descriptors, which in the lower approximation, the possible decision rules can be generated from the descriptors, which are in the boundary region. Therefore, to obtain the simplified decision rules, the concept of reduct can also be introduced into the lower approximation and boundary region.

**Definition 20 .**

Let *S* = (*U*, AT ∪ {*d*}) = (*U*, {*a*_*j*_^*k*^ : *k* = 1,2,…, *I*, *j* = 1,2,…, *m*}∪{*d*}) be an incomplete multiscale decision system in which *t* ^*k*^ ∈ FDES(AT^*k*^), *t* ^′*k*^ ∈ DES(AT^*k*^), and *t* ^′*k*^⪰*t* ^*k*^; define

(25) tLk={Xi∈UIND({d}):||tk||⊆Xi},tBk={Xi∈UIND({d}):||tk||∩Xi≠∅∧||tk||⊈Xi}.

Then *t* ^′*k*^ is referred to as the lower approximation reduct descriptor of *t* ^*k*^ if and only if the following two conditions hold:

1. *t*_*L*_^*k*^ = *t*_*L*_^′*k*^;
2. *t*_*L*_^′′*k*^ ≠ *t*_*L*_^*k*^ for each *t* ^′′*k*^≻*t* ^′*k*^;

*t* ^′*k*^ is referred to as the boundary region reduct descriptor of *t* ^*k*^ if and only if the following two conditions hold:

1. *t*_*B*_^*k*^ = *t*_*B*_^′*k*^;
2. *t*_*B*_^′′*k*^ ≠ *t*_*B*_^*k*^ for each *t* ^′′*k*^≻*t* ^′*k*^.

By Definition 20, we can see that a lower approximation reduct descriptor of *t* ^*k*^ is a minimal conjunction of the atomic properties in *t* ^*k*^, which preserves the inclusion relation between support of *t* ^*k*^ and the decision classes; a boundary region reduct descriptor of *t* ^*k*^ is a minimal conjunction of the atomic properties in *t* ^*k*^, which preserves the intersection relation between support of *t* ^*k*^ and the decision classes.

**Remark 21 .**

It should be noticed that if *t*_*L*_^*k*^ = *∅*, then ||*t* ^*k*^||⊈*X*_*i*_ for each *X*_*i*_ ∈ *U*/IND({*d*}); it follows that no certain decision rules are supported by the *k*-scale descriptor *t* ^*k*^. Therefore, it is meaningless to compute the lower approximation reduct descriptor of *t* ^*k*^ if *t*_*L*_^*k*^ = *∅*.

Given an incomplete multiscale decision system *S* = (*U*, AT ∪ {*d*}) = (*U*, {*a*_*j*_^*k*^ : *k* = 1,2,…, *I*, *j* = 1,2,…, *m*}∪{*d*}), if *t*_*L*_^*k*^ = {*X*_*i*_}, then ||*t* ^*k*^||⊆*X*_*i*_; we can obtain a certain decision rule such that *r* ^*k*^ : *t* ^*k*^ → *X*_*i*_. Moreover, suppose that *t* ^′*k*^ is a lower approximation reduct descriptor of *t* ^*k*^; then *t* ^′*k*^ is a minimal conjunction of the atomic properties in *t* ^*k*^, which preserves the inclusion property; therefore, the decision rule *r* ^*k*^ : *t* ^*k*^ → *X*_*i*_ can be simplified to be *r* ^′*k*^ : *t* ^′*k*^ → *X*_*i*_. The condition part of *r* ^*k*^ : *t* ^*k*^ → *X*_*i*_ is shortened because *t* ^′*k*^⪰*t* ^*k*^. Similar to what have been analyzed, by the boundary region reduct descriptor, the possible rules supported by *t* ^*k*^ can also be simplified.

To compute the lower approximation and boundary region reduct descriptor of *t* ^*k*^, let us define

(26) DISL(tk,y)={{ajk∈ATk:y∉||(ajk,vjk)||, (ajk,vjk)∈tk}:||tk||⊆Xi,  y∉Xi,∅:otherwise;DISB(tk,y)={{ajk∈ATk:y∉||(ajk,vjk)||, (ajk,vjk)∈tk}:||tk||∩Xi=∅,  y∈Xi,∅:otherwise

then

(27) MLtk={DISL(tk,y):||tk||⊆Xi,y∉Xi},MBtk={DISL(tk,y):||tk||∩Xi=∅,y∈Xi}

are referred to as the *t* ^*k*^ lower approximation and *t* ^*k*^ boundary region discernibility matrixes, respectively.

**Theorem 22 .**

Let *S* = (*U*, *AT* ∪ {*d*}) = (*U*, {*a*_*j*_^*k*^ : *k* = 1,2,…, *I*, *j* = 1,2,…, *m*}∪{*d*}) be an incomplete multiscale decision system in which *t* ^*k*^ ∈ *FDES*(*AT* ^*k*^), *t* ^′*k*^ ∈ *DES*(*AT* ^*k*^), and *t* ^′*k*^⪰*t* ^*k*^:

1. *t*_*L*_^*k*^ = *t*_*L*_^′*k*^⇔*AT* ^*k*^(*t* ^′*k*^)∩*DIS*_*L*_(*t* ^*k*^, *y*) ≠ *∅* for each *DIS*_*L*_(*t* ^*k*^, *y*) ∈ **M**_*L*_^*t*^*k*^^;
2. *t*_*B*_^*k*^ = *t*_*B*_^′*k*^⇔*AT* ^*k*^(*t* ^′*k*^)∩*DIS*_*B*_(*t* ^*k*^, *y*) ≠ *∅* for each *DIS*_*B*_(*t* ^*k*^, *y*) ∈ **M**_*B*_^*t*^*k*^^.

**Proof**

We only prove (1); the proof of (2) is similar to the proof of (1).

⇒: Suppose ∃DIS_*L*_(*t* ^*k*^, *y*) ∈ **M**_*L*_^*t*^*k*^^ such that AT^*k*^(*t* ^′*k*^)∩DIS_*L*_(*t* ^*k*^, *y*) = *∅*; then we have *y* ∈ ||*t* ^′*k*^|| because *t* ^′*k*^⪰*t* ^*k*^. Moreover, by condition we have *t*_*L*_^*k*^ = *t*_*L*_^′*k*^; that is, ||*t* ^*k*^||⊆*X*_*i*_⇔||*t* ^′*k*^||⊆*X*_*i*_; we know *y* ∈ *X*_*i*_; such result is contradictive to the assumption because DIS_*L*_(*t* ^*k*^, *y*) ∈ **M**_*L*_^*t*^*k*^^⇒*y* ∉ *X*_*i*_.

⇐: Since *t* ^′*k*^⪰*t* ^*k*^, then ||*t* ^′*k*^||⊇||*t* ^*k*^||, from which we obtain *t*_*L*_^*k*^⊇*t*_*L*_^′*k*^. Thus, it must be proved that *t*_*L*_^*k*^⊆*t*_*L*_^′*k*^. ∀*X*_*i*_ ∈ *t*_*L*_^*k*^, we have ||*t* ^*k*^||⊆*X*_*i*_. Then ∀*y* ∉ *X*_*i*_, we have DIS_*L*_(*t* ^*k*^, *y*) ∈ **M**_*L*_^*t*^*k*^^; by condition we know that AT^*k*^(*t* ^′*k*^)∩DIS_*L*_(*t* ^*k*^, *y*) ≠ *∅*; it follows that *y* ∉ ||*t*′||; then there must be *a*_*j*_^*k*^ ∈ AT^*k*^(*t* ^′*k*^) such that *y* ∉ ||*a*_*j*_^*k*^, *v*_*j*_^*k*^||; it follows that *y* ∉ ||*t* ^′*k*^||, from which we can conclude that ||*t* ^′*k*^||⊆*X*_*i*_; that is, *X*_*i*_ ∈ *t*_*L*_^′*k*^, *t*_*L*_^*k*^⊇*t*_*L*_^′*k*^. That completes the proof.

**Definition 23 .**

Let *S* = (*U*, AT ∪ {*d*}) = (*U*, {*a*_*j*_^*k*^ : *k* = 1,2,…, *I*, *j* = 1,2,…, *m*}∪{*d*}) be an incomplete multiscale decision system in which *t* ^*k*^ ∈ FDES(AT^*k*^); then

(28) $$\begin{array}{c} \Delta_{L}^{t^{k}}=\overset{}{\underset{{\text{DIS}}_{L}(t^{k},y)\in M_{L}^{t^{k}}}{⋀}}\overset{}{\underset{}{⋁}}{\text{DIS}}_{L}\left(t^{k},y\right) \end{array}$$

is referred to as the *t* ^*k*^ lower approximation discernibility function;

(29) $$\begin{array}{c} \Delta_{B}^{t^{k}}=\overset{}{\underset{{\text{DIS}}_{B}(t^{k},y)\in M_{B}^{t^{k}}}{⋀}}\overset{}{\underset{}{⋁}}{\text{DIS}}_{B}\left(t^{k},y\right) \end{array}$$

is referred to as the *t* ^*k*^ boundary region discernibility function.

**Theorem 24 .**

Let *S* = (*U*, *AT* ∪ {*d*}) = (*U*, {*a*_*j*_^*k*^ : *k* = 1,2,…, *I*, *j* = 1,2,…, *m*}∪{*d*}) be an incomplete multiscale decision system in which *t* ^*k*^ ∈ *FDES*(*AT* ^*k*^), *t* ^′*k*^ ∈ *DES*(*AT* ^*k*^), and *t* ^′*k*^⪰*t* ^*k*^; then

1. *t* ^′*k*^ is a lower approximation reduct descriptor of *t* ^*k*^ if and only if *AT* ^*k*^(*t* ^′*k*^) is the prime implicant of the *t* ^*k*^ lower approximation discernibility function Δ_*L*_^*t*^*k*^^;
2. *t* ^′*k*^ is a boundary region reduct descriptor of *t* ^*k*^ if and only if *AT* ^*k*^(*t* ^′*k*^) is the prime implicant of the *t* ^*k*^ boundary region discernibility function Δ_*B*_^*t*^*k*^^.

**Proof**

The proof of Theorem 24 is similar to the proof of Theorem 19.

**Example 25 .**

In Table 1, if the support of a descriptor is in the lower approximation of a decision class, then we can compute the lower approximation reduct descriptor to derive the simplified certain decision rule; similarly, if the support of a descriptor is in the boundary region of a decision class, then we can compute the boundary region reduct descriptor to derive the simplified possible decision rule.

By the discernibility matrixes we mentioned above, it is not difficult to obtain the lower approximation and boundary region reduct descriptors as Tables 5 and 6 show, respectively.

By the lower approximation reduct descriptors shown in Table 5, we can derive the following certain decision rules:

1. * 1-scale certain decision rules:*
  - *a*_1_^1^(*x*) = 1,4, 5,7, 8∧*a*_2_^1^(*x*) = 3 → *d*(*x*) = 1,
  - *a*_1_^1^(*x*) = 1,3, 4,5, 7,8∧*a*_3_^1^(*x*) = 5 → *d*(*x*) = 1,
  - *a*_1_^1^(*x*) = 1,4, 5,7, 8∧*a*_4_^1^(*x*) = 2 → *d*(*x*) = 1,
  - *a*_1_^1^(*x*) = 1,2, 3,5, 6,7, 8∧*a*_3_^1^(*x*) = 3 → *d*(*x*) = 2,
  - *a*_1_^1^(*x*) = 2,3, 5,6, 7,8∧*a*_2_^1^(*x*) = 1 → *d*(*x*) = 2,
  - *a*_1_^1^(*x*) = 3,6, 7,8∧*a*_4_^1^(*x*) = 1 → *d*(*x*) = 2,
  - *a*_2_^1^(*x*) = 4,5, 6 → *d*(*x*) = 2,
  - *a*_1_^1^ = 4∧*a*_3_^1^ = 1 → *d*(*x*) = 2,
  - *a*_4_^1^(*x*) = 3,4 → *d*(*x*) = 2,
  - *a*_2_^1^(*x*) = 7∨*a*_3_^1^(*x*) = 6∨*a*_4_^1^(*x*) = 5,6 → *d*(*x*) = 3;
2. * 2-scale certain decision rules:*
  - *a*_1_^2^(*x*) = *F*∧*a*_3_^2^(*x*) = *L*, *H* → *d*(*x*) = 1,
  - *a*_3_^2^(*x*) = *H*∧*a*_4_^2^(*x*) = *L* → *d*(*x*) = 1,
  - *a*_4_^2^(*x*) = *M* → *d*(*x*) = 2,
  - *a*_1_^2^(*x*) = *F*∧*a*_2_^2^(*x*) = *F*, *B* → *d*(*x*) = 2,
  - *a*_2_^2^(*x*) = *F*, *B*∧*a*_3_^2^(*x*) = *M* → *d*(*x*) = 2,
  - *a*_2_^2^(*x*) = *F*∧*a*_4_^2^(*x*) = *L* → *d*(*x*) = 2,
  - *a*_1_^2^(*x*) = *B*∨*a*_4_^2^(*x*) = *H* → *d*(*x*) = 3,
  - *a*_2_^2^(*x*) = *B*, *F*∧*a*_3_^2^(*x*) = *H* → *d*(*x*) = 3;
3. * 3-scale certain decision rules:*
  - *a*_2_^3^(*x*) = *N*∧*a*_4_^3^(*x*) = *Y* → *d*(*x*) = 2.

By the boundary region reduct descriptors shown in Table 6, we can derive the following possible decision rules:

1. * 1-scale possible decision rules:*
  - *a*_1_^1^(*x*) = 1∧*a*_3_^1^(*x*) = 1 → *d*(*x*) = 1∨3 with certainty 0.5,
  - *a*_2_^1^(*x*) = 1∧*a*_3_^1^(*x*) = 1 → *d*(*x*) = 1∨3 with certainty 0.5,
  - *a*_1_^1^(*x*) = 2∧*a*_2_^1^(*x*) = 2 → *d*(*x*) = 1∨3 with certainty 0.5,
  - *a*_1_^1^(*x*) = 2∧*a*_3_^1^(*x*) = 1 → *d*(*x*) = 1∨3 with certainty 0.5,
  - *a*_1_^1^(*x*) = 3∧*a*_2_^1^(*x*) = 3 → *d*(*x*) = 1 with certainty 0.67,
  - *a*_1_^1^(*x*) = 3∧*a*_2_^1^(*x*) = 3 → *d*(*x*) = 3 with certainty 0.33,
  - *a*_3_^1^(*x*) = 2 → *d*(*x*) = 1∨3 with certainty 0.5,
  - *a*_1_^1^(*x*) = 3∧*a*_4_^1^(*x*) = 2 → *d*(*x*) = 1 with certainty 0.67,
  - *a*_1_^1^(*x*) = 3∧*a*_4_^1^(*x*) = 2 → *d*(*x*) = 3 with certainty 0.33,
  - *a*_1_^1^(*x*) = 4∧*a*_3_^1^(*x*) = 3 → *d*(*x*) = 2 with certainty 0.67,
  - *a*_1_^1^(*x*) = 4∧*a*_3_^1^(*x*) = 3 → *d*(*x*) = 1 with certainty 0.33,
  - *a*_1_^1^(*x*) = 5∧*a*_3_^1^(*x*) = 4 → *d*(*x*) = 2 with certainty 0.75,
  - *a*_1_^1^(*x*) = 5∧*a*_3_^1^(*x*) = 4 → *d*(*x*) = 1 with certainty 0.25,
  - *a*_1_^1^(*x*) = 6∧*a*_3_^1^(*x*) = 5 → *d*(*x*) = 2 with certainty 0.75,
  - *a*_1_^1^(*x*) = 6∧*a*_3_^1^(*x*) = 5 → *d*(*x*) = 1 with certainty 0.25;
2. * 2-scale possible decision rules:*
  - *a*_1_^2^ = *G*∧*a*_3_^2^ = *L* → *d*(*x*) = 1∨3 with certainty 0.5,
  - *a*_1_^2^ = *F*∧*a*_3_^2^ = *M* → *d*(*x*) = 2 with certainty 0.75,
  - *a*_1_^2^ = *F*∧*a*_3_^2^ = *M* → *d*(*x*) = 1 with certainty 0.25;
3. * 3-scale possible decision rules:*
  - *a*_1_^3^ = *Y*∨*a*_3_^3^ = *Y* → *d*(*x*) = 1∨3 with certainty 0.5,
  - *a*_1_^3^ = *N*∨*a*_2_^3^ = *Y* → *d*(*x*) = 1∨2 with certainty 0.5,
  - *a*_1_^3^ = *N*∨*a*_4_^3^ = *Y* → *d*(*x*) = 2 with certainty 0.67,
  - *a*_1_^3^ = *N*∨*a*_4_^3^ = *Y* → *d*(*x*) = 1 with certainty 0.33,
  - *a*_2_^3^ = *Y*∨*a*_3_^3^ = *N* → *d*(*x*) = 1∨2 with certainty 0.5,
  - *a*_3_^3^ = *N*∨*a*_4_^3^ = *Y* → *d*(*x*) = 2 with certainty 0.67,
  - *a*_3_^3^ = *N*∨*a*_4_^3^ = *Y* → *d*(*x*) = 1 with certainty 0.33,
  - *a*_2_^3^ = *N* → *d*(*x*) = 2 with certainty 0.75,
  - *a*_2_^3^ = *N* → *d*(*x*) = 3 with certainty 0.25,
  - *a*_4_^3^ = *N* → *d*(*x*) = 2 with certainty 0.67,
  - *a*_4_^3^ = *N* → *d*(*x*) = 3 with certainty 0.33.

## 5. Conclusion

In this paper, the incomplete multiscale information system is explored through the well-known rough set approach. The incomplete multiscale information system is different from the traditional incomplete information system since it reflects data represented at different levels of granulations, which are transformed from smaller to bigger scales. At each level of granulation, that is, scale, we can construct the corresponding rough set and then derive decision rules. Obviously, the decision rules derived from a coarser scale are more general than that from a finer scale. The incomplete multiscale information system is closely related to multigranulation rough set approach. The rough set at the smallest scale is optimistic multigranulation rough set while the rough set at the biggest scale is pessimistic multigranulation rough set.

Obviously, the study of multiscale information system will give new life to the development of rough set theory. Furthermore, by considering the preference-ordered [45–47] domains of the attributes, new approaches to granular representations and new models for knowledge acquisition in multiscale frame are an interesting topic to be addressed.

## Figures and Tables

**Table 1:** An example of incomplete multiscale decision system.

*U*	*a*_1_^1^	*a*_1_^2^	*a*_1_^3^	*a*_2_^1^	*a*_2_^2^	*a*_2_^3^	*a*_3_^1^	*a*_3_^2^	*a*_3_^3^	*a*_4_^1^	*a*_4_^2^	*a*_4_^3^	*d*
*x*_1_	1	*G*	*Y*	1	*G*	*Y*	1	*L*	*Y*	1	*L*	*Y*	1
*x*_2_	2	*G*	*Y*	2	*G*	*Y*	1	*L*	*Y*	1	*L*	*Y*	1
*x*_3_	3	*G*	*Y*	3	*G*	*Y*	2	*L*	*Y*	2	*L*	*Y*	1
*x*_4_	4	*F*	*N*	4	*F*	*N*	3	*M*	*N*	3	*M*	*Y*	2
*x*_5_	5	*F*	*N*	5	∗	*N*	4	*M*	*N*	4	*M*	*N*	2
*x*_6_	6	*F*	*N*	6	*F*	*N*	5	*M*	*N*	4	*M*	*N*	2
*x*_7_	∗	*F*	*N*	1	*G*	*Y*	3	*M*	*N*	1	*L*	*N*	2
*x*_8_	5	*F*	*N*	2	*G*	*Y*	4	*M*	*N*	1	*L*	*Y*	2
*x*_9_	6	*F*	*N*	3	*G*	*Y*	5	*M*	*N*	2	*L*	*Y*	2
*x*_10_	4	*F*	*N*	4	*F*	*N*	1	*M*	*N*	1	*L*	∗	2
*x*_11_	5	*F*	*N*	5	*F*	*N*	4	*M*	*N*	1	*L*	*Y*	2
*x*_12_	6	*F*	*N*	6	*F*	*N*	5	*M*	*N*	2	*L*	*Y*	2
*x*_13_	4	*F*	*N*	1	*G*	*Y*	3	*M*	*N*	1	*L*	*Y*	1
*x*_14_	5	*F*	*N*	2	*G*	*Y*	4	*M*	*N*	1	*L*	*Y*	1
*x*_15_	∗	*F*	*N*	3	*G*	*Y*	5	∗	*N*	2	*L*	*Y*	1
*x*_16_	1	*G*	*Y*	1	*G*	*Y*	1	*L*	*Y*	1	*L*	*Y*	3
*x*_17_	2	*G*	*Y*	2	*G*	*Y*	1	*L*	*Y*	1	*L*	*Y*	3
*x*_18_	3	*G*	*Y*	3	*G*	*Y*	2	*L*	*Y*	2	*L*	*Y*	3
*x*_19_	7	*B*	*N*	7	*B*	*N*	6	*H*	*N*	5	*H*	*N*	3
*x*_20_	8	*B*	*N*	7	∗	*N*	6	*H*	*N*	6	*H*	*N*	3

**Table 2:** Full AT^1^ descriptors and their supports.

Descriptors	Supports
*t*_1_^1^ = (*a*_1_^1^, 1)∧(*a*_2_^1^, 1)∧(*a*_3_^1^, 1)∧(*a*_4_^1^, 1)	||*t*_1_^1^ | | = {*x*_1_, *x*_16_}
*t*_2_^1^ = (*a*_1_^1^, 1)∧(*a*_2_^1^, 1)∧(*a*_3_^1^, 3)∧(*a*_4_^1^, 1)	||*t*_2_^1^ | | = {*x*_7_}
*t*_3_^1^ = (*a*_1_^1^, 1)∧(*a*_2_^1^, 3)∧(*a*_3_^1^, 5)∧(*a*_4_^1^, 2)	||*t*_3_^1^ | | = {*x*_15_}
*t*_4_^1^ = (*a*_1_^1^, 2)∧(*a*_2_^1^, 1)∧(*a*_3_^1^, 3)∧(*a*_4_^1^, 1)	||*t*_4_^1^ | | = {*x*_7_}
*t*_5_^1^ = (*a*_1_^1^, 2)∧(*a*_2_^1^, 2)∧(*a*_3_^1^, 1)∧(*a*_4_^1^, 1)	||*t*_5_^1^ | | = {*x*_2_, *x*_17_}
*t*_6_^1^ = (*a*_1_^1^, 3)∧(*a*_2_^1^, 1)∧(*a*_3_^1^, 3)∧(*a*_4_^1^, 1)	||*t*_6_^1^ | | = {*x*_7_}
*t*_7_^1^ = (*a*_1_^1^, 3)∧(*a*_2_^1^, 3)∧(*a*_3_^1^, 2)∧(*a*_4_^1^, 2)	||*t*_7_^1^ | | = {*x*_3_, *x*_18_}
*t*_8_^1^ = (*a*_1_^1^, 3)∧(*a*_2_^1^, 3)∧(*a*_3_^1^, 5)∧(*a*_4_^1^, 2)	||*t*_8_^1^ | | = {*x*_15_}
*t*_9_^1^ = (*a*_1_^1^, 4)∧(*a*_2_^1^, 1)∧(*a*_3_^1^, 3)∧(*a*_4_^1^, 1)	||*t*_9_^1^ | | = {*x*_7_, *x*_13_}
*t*_10_^1^ = (*a*_1_^1^, 4)∧(*a*_2_^1^, 3)∧(*a*_3_^1^, 5)∧(*a*_4_^1^, 2)	||*t*_10_^1^ | | = {*x*_15_}
*t*_11_^1^ = (*a*_1_^1^, 4)∧(*a*_2_^1^, 4)∧(*a*_3_^1^, 1)∧(*a*_4_^1^, 1)	||*t*_11_^1^ | | = {*x*_10_}
*t*_12_^1^ = (*a*_1_^1^, 4)∧(*a*_2_^1^, 4)∧(*a*_3_^1^, 3)∧(*a*_4_^1^, 3)	||*t*_12_^1^ | | = {*x*_4_}
*t*_13_^1^ = (*a*_1_^1^, 5)∧(*a*_2_^1^, 1)∧(*a*_3_^1^, 3)∧(*a*_4_^1^, 1)	||*t*_13_^1^ | | = {*x*_7_}
*t*_14_^1^ = (*a*_1_^1^, 5)∧(*a*_2_^1^, 2)∧(*a*_3_^1^, 4)∧(*a*_4_^1^, 1)	||*t*_14_^1^ | | = {*x*_8_, *x*_14_}
*t*_15_^1^ = (*a*_1_^1^, 5)∧(*a*_2_^1^, 3)∧(*a*_3_^1^, 5)∧(*a*_4_^1^, 2)	||*t*_15_^1^ | | = {*x*_15_}
*t*_16_^1^ = (*a*_1_^1^, 5)∧(*a*_2_^1^, 5)∧(*a*_3_^1^, 4)∧(*a*_4_^1^, 1)	||*t*_16_^1^ | | = {*x*_11_}
*t*_17_^1^ = (*a*_1_^1^, 5)∧(*a*_2_^1^, 5)∧(*a*_3_^1^, 4)∧(*a*_4_^1^, 4)	||*t*_17_^1^ | | = {*x*_5_}
*t*_18_^1^ = (*a*_1_^1^, 6)∧(*a*_2_^1^, 1)∧(*a*_3_^1^, 3)∧(*a*_4_^1^, 1)	||*t*_18_^1^ | | = {*x*_7_}
*t*_19_^1^ = (*a*_1_^1^, 6)∧(*a*_2_^1^, 3)∧(*a*_3_^1^, 5)∧(*a*_4_^1^, 2)	||*t*_19_^1^ | | = {*x*_9_, *x*_15_}
*t*_20_^1^ = (*a*_1_^1^, 6)∧(*a*_2_^1^, 6)∧(*a*_3_^1^, 5)∧(*a*_4_^1^, 4)	||*t*_20_^1^ | | = {*x*_6_}
*t*_21_^1^ = (*a*_1_^1^, 6)∧(*a*_2_^1^, 6)∧(*a*_3_^1^, 5)∧(*a*_4_^1^, 2)	||*t*_21_^1^ | | = {*x*_12_}
*t*_22_^1^ = (*a*_1_^1^, 7)∧(*a*_2_^1^, 1)∧(*a*_3_^1^, 3)∧(*a*_4_^1^, 1)	||*t*_22_^1^ | | = {*x*_7_}
*t*_23_^1^ = (*a*_1_^1^, 7)∧(*a*_2_^1^, 3)∧(*a*_3_^1^, 5)∧(*a*_4_^1^, 2)	||*t*_23_^1^ | | = {*x*_15_}
*t*_24_^1^ = (*a*_1_^1^, 7)∧(*a*_2_^1^, 7)∧(*a*_3_^1^, 6)∧(*a*_4_^1^, 5)	||*t*_24_^1^ | | = {*x*_19_}
*t*_25_^1^ = (*a*_1_^1^, 8)∧(*a*_2_^1^, 1)∧(*a*_3_^1^, 3)∧(*a*_4_^1^, 1)	||*t*_25_^1^ | | = {*x*_7_}
*t*_26_^1^ = (*a*_1_^1^, 8)∧(*a*_2_^1^, 3)∧(*a*_3_^1^, 5)∧(*a*_4_^1^, 2)	||*t*_26_^1^ | | = {*x*_15_}
*t*_27_^1^ = (*a*_1_^1^, 8)∧(*a*_2_^1^, 7)∧(*a*_3_^1^, 6)∧(*a*_4_^1^, 6)	||*t*_27_^1^ | | = {*x*_20_}

**Table 3:** Full AT^2^ descriptors and their supports.

Descriptors	Supports
*t*_1_^2^ = (*a*_1_^2^, *G*)∧(*a*_2_^2^, *G*)∧(*a*_3_^2^, *L*)∧(*a*_4_^2^, *L*)	||*t*_1_^2^ | | = {*x*_1_, *x*_2_, *x*_3_, *x*_16_, *x*_17_, *x*_18_}
*t*_2_^2^ = (*a*_1_^2^, *F*)∧(*a*_2_^2^, *G*)∧(*a*_3_^2^, *M*)∧(*a*_4_^2^, *M*)	||*t*_2_^2^ | | = {*x*_5_}
*t*_3_^2^ = (*a*_1_^2^, *F*)∧(*a*_2_^2^, *G*)∧(*a*_3_^2^, *M*)∧(*a*_4_^2^, *L*)	||*t*_3_^2^ | | = {*x*_7_, *x*_8_, *x*_9_, *x*_13_, *x*_14_, *x*_15_}
*t*_4_^2^ = (*a*_1_^2^, *F*)∧(*a*_2_^2^, *F*)∧(*a*_3_^2^, *M*)∧(*a*_4_^2^, *M*)	||*t*_4_^2^ | | = {*x*_4_, *x*_5_, *x*_6_}
*t*_5_^2^ = (*a*_1_^2^, *F*)∧(*a*_2_^2^, *F*)∧(*a*_3_^2^, *M*)∧(*a*_4_^2^, *L*)	||*t*_5_^2^ | | = {*x*_10_, *x*_11_, *x*_12_}
*t*_6_^2^ = (*a*_1_^2^, *F*)∧(*a*_2_^2^, *B*)∧(*a*_3_^2^, *M*)∧(*a*_4_^2^, *M*)	||*t*_6_^2^ | | = {*x*_5_}
*t*_7_^2^ = (*a*_1_^2^, *F*)∧(*a*_2_^2^, *G*)∧(*a*_3_^2^, *L*)∧(*a*_4_^2^, *L*)	||*t*_7_^2^ | | = {*x*_15_}
*t*_8_^2^ = (*a*_1_^2^, *F*)∧(*a*_2_^2^, *G*)∧(*a*_3_^2^, *H*)∧(*a*_4_^2^, *L*)	||*t*_8_^2^ | | = {*x*_15_}
*t*_9_^2^ = (*a*_1_^2^, *B*)∧(*a*_2_^2^, *B*)∧(*a*_3_^2^, *H*)∧(*a*_4_^2^, *H*)	||*t*_9_^2^ | | = {*x*_19_, *x*_20_}
*t*_10_^2^ = (*a*_1_^2^, *B*)∧(*a*_2_^2^, *F*)∧(*a*_3_^2^, *H*)∧(*a*_4_^2^, *H*)	||*t*_10_^2^ | | = {*x*_20_}
*t*_11_^2^ = (*a*_1_^2^, *B*)∧(*a*_2_^2^, *G*)∧(*a*_3_^2^, *H*)∧(*a*_4_^2^, *H*)	||*t*_11_^2^ | | = {*x*_20_}

**Table 4:** Full AT^3^ descriptors and their supports.

Descriptors	Supports
*t*_1_^3^ = (*a*_1_^3^, *Y*)∧(*a*_2_^3^, *Y*)∧(*a*_3_^3^, *Y*)∧(*a*_4_^3^, *Y*)	||*t*_1_^3^ | | = {*x*_1_, *x*_2_, *x*_3_, *x*_16_, *x*_17_, *x*_18_}
*t*_2_^3^ = (*a*_1_^3^, *N*)∧(*a*_2_^3^, *Y*)∧(*a*_3_^3^, *N*)∧(*a*_4_^3^, *Y*)	||*t*_2_^3^ | | = {*x*_7_, *x*_8_, *x*_9_, *x*_13_, *x*_14_, *x*_15_}
*t*_3_^3^ = (*a*_1_^3^, *N*)∧(*a*_2_^3^, *N*)∧(*a*_3_^3^, *N*)∧(*a*_4_^3^, *N*)	||*t*_3_^3^ | | = {*x*_4_, *x*_5_, *x*_6_, *x*_10_, *x*_19_, *x*_20_}
*t*_4_^3^ = (*a*_1_^3^, *N*)∧(*a*_2_^3^, *N*)∧(*a*_3_^3^, *N*)∧(*a*_4_^3^, *Y*)	||*t*_4_^3^ | | = {*x*_10_, *x*_11_, *x*_12_}

**Table 5:** Lower approximation reduct descriptors.

Original descriptors	Lower approximation reduct descriptors
*t*_3_^1^ = (*a*_1_^1^, 1)∧(*a*_2_^1^, 3)∧(*a*_3_^1^, 5)∧(*a*_4_^1^, 2)	(*a*_1_^1^, 1)∧(*a*_2_^1^, 3), (*a*_1_^1^, 1)∧(*a*_3_^1^, 5), (*a*_1_^1^, 1)∧(*a*_4_^1^, 2)
*t*_8_^1^ = (*a*_1_^1^, 3)∧(*a*_2_^1^, 3)∧(*a*_3_^1^, 5)∧(*a*_4_^1^, 2)	(*a*_1_^1^, 3)∧(*a*_3_^1^, 5)
*t*_10_^1^ = (*a*_1_^1^, 4)∧(*a*_2_^1^, 3)∧(*a*_3_^1^, 5)∧(*a*_4_^1^, 2)	(*a*_1_^1^, 4)∧(*a*_2_^1^, 3), (*a*_1_^1^, 4)∧(*a*_3_^1^, 5), (*a*_1_^1^, 4)∧(*a*_4_^1^, 2)
*t*_15_^1^ = (*a*_1_^1^, 5)∧(*a*_2_^1^, 3)∧(*a*_3_^1^, 5)∧(*a*_4_^1^, 2)	(*a*_1_^1^, 5)∧(*a*_2_^1^, 3), (*a*_1_^1^, 5)∧(*a*_3_^1^, 5), (*a*_1_^1^, 5)∧(*a*_4_^1^, 2)
*t*_23_^1^ = (*a*_1_^1^, 7)∧(*a*_2_^1^, 3)∧(*a*_3_^1^, 5)∧(*a*_4_^1^, 2)	(*a*_1_^1^, 7)∧(*a*_2_^1^, 3), (*a*_1_^1^, 7)∧(*a*_3_^1^, 5), (*a*_1_^1^, 7)∧(*a*_4_^1^, 2)
*t*_26_^1^ = (*a*_1_^1^, 8)∧(*a*_2_^1^, 3)∧(*a*_3_^1^, 5)∧(*a*_4_^1^, 2)	(*a*_1_^1^, 8)∧(*a*_2_^1^, 3), (*a*_1_^1^, 8)∧(*a*_3_^1^, 5), (*a*_1_^1^, 8)∧(*a*_4_^1^, 2)
*t*_2_^1^ = (*a*_1_^1^, 1)∧(*a*_2_^1^, 1)∧(*a*_3_^1^, 3)∧(*a*_4_^1^, 1)	(*a*_1_^1^, 1)∧(*a*_3_^1^, 3)
*t*_4_^1^ = (*a*_1_^1^, 2)∧(*a*_2_^1^, 1)∧(*a*_3_^1^, 3)∧(*a*_4_^1^, 1)	(*a*_1_^1^, 2)∧(*a*_2_^1^, 1), (*a*_1_^1^, 2)∧(*a*_3_^1^, 3)
*t*_6_^1^ = (*a*_1_^1^, 3)∧(*a*_2_^1^, 1)∧(*a*_3_^1^, 3)∧(*a*_4_^1^, 1)	(*a*_1_^1^, 3)∧(*a*_2_^1^, 1), (*a*_1_^1^, 3)∧(*a*_3_^1^, 3), (*a*_1_^1^, 3)∧(*a*_4_^1^, 1)
*t*_11_^1^ = (*a*_1_^1^, 4)∧(*a*_2_^1^, 4)∧(*a*_3_^1^, 1)∧(*a*_4_^1^, 1)	(*a*_2_^1^, 4), (*a*_1_^1^, 4)∧(*a*_3_^1^, 1)
*t*_12_^1^ = (*a*_1_^1^, 4)∧(*a*_2_^1^, 4)∧(*a*_3_^1^, 3)∧(*a*_4_^1^, 3)	(*a*_2_^1^, 4), (*a*_4_^1^, 3)
*t*_13_^1^ = (*a*_1_^1^, 5)∧(*a*_2_^1^, 1)∧(*a*_3_^1^, 3)∧(*a*_4_^1^, 1)	(*a*_1_^1^, 5)∧(*a*_2_^1^, 1), (*a*_1_^1^, 5)(*a*_3_^1^, 3)
*t*_16_^1^ = (*a*_1_^1^, 5)∧(*a*_2_^1^, 5)∧(*a*_3_^1^, 4)∧(*a*_4_^1^, 1)	(*a*_2_^1^, 5)
*t*_17_^1^ = (*a*_1_^1^, 5)∧(*a*_2_^1^, 5)∧(*a*_3_^1^, 4)∧(*a*_4_^1^, 4)	(*a*_2_^1^, 5), (*a*_4_^1^, 4)
*t*_18_^1^ = (*a*_1_^1^, 6)∧(*a*_2_^1^, 1)∧(*a*_3_^1^, 3)∧(*a*_4_^1^, 1)	(*a*_1_^1^, 6)∧(*a*_2_^1^, 1), (*a*_1_^1^, 6)∧(*a*_3_^1^, 3), (*a*_1_^1^, 6)∧(*a*_4_^1^, 1)
*t*_20_^1^ = (*a*_1_^1^, 6)∧(*a*_2_^1^, 6)∧(*a*_3_^1^, 5)∧(*a*_4_^1^, 4)	(*a*_2_^1^, 6), (*a*_4_^1^, 4)
*t*_21_^1^ = (*a*_1_^1^, 6)∧(*a*_2_^1^, 6)∧(*a*_3_^1^, 5)∧(*a*_4_^1^, 2)	(*a*_2_^1^, 6)
*t*_22_^1^ = (*a*_1_^1^, 7)∧(*a*_2_^1^, 1)∧(*a*_3_^1^, 3)∧(*a*_4_^1^, 1)	(*a*_1_^1^, 7)∧(*a*_2_^1^, 1), (*a*_1_^1^, 7)∧(*a*_3_^1^, 3), (*a*_1_^1^, 7)∧(*a*_4_^1^, 1)
*t*_25_^1^ = (*a*_1_^1^, 8)∧(*a*_2_^1^, 1)∧(*a*_3_^1^, 3)∧(*a*_4_^1^, 1)	(*a*_1_^1^, 8)∧(*a*_2_^1^, 1), (*a*_1_^1^, 8)∧(*a*_3_^1^, 3), (*a*_1_^1^, 8)∧(*a*_4_^1^, 1)
*t*_24_^1^ = (*a*_1_^1^, 7)∧(*a*_2_^1^, 7)∧(*a*_3_^1^, 6)∧(*a*_4_^1^, 5)	(*a*_2_^1^, 7), (*a*_3_^1^, 6), (*a*_4_^1^, 5)
*t*_27_^1^ = (*a*_1_^1^, 8)∧(*a*_2_^1^, 7)∧(*a*_3_^1^, 6)∧(*a*_4_^1^, 6)	(*a*_2_^1^, 7), (*a*_3_^1^, 6), (*a*_4_^1^, 6)
*t*_7_^2^ = (*a*_1_^2^, *F*)∧(*a*_2_^2^, *G*)∧(*a*_3_^2^, *L*)∧(*a*_4_^2^, *L*)	(*a*_1_^2^, *F*)∧(*a*_3_^2^, *L*)
*t*_8_^2^ = (*a*_1_^2^, *F*)∧(*a*_2_^2^, *G*)∧(*a*_3_^2^, *H*)∧(*a*_4_^2^, *L*)	(*a*_1_^2^, *F*)∧(*a*_3_^2^, *H*), (*a*_3_^2^, *H*)∧(*a*_4_^2^, *L*)
*t*_2_^2^ = (*a*_1_^2^, *F*)∧(*a*_2_^2^, *G*)∧(*a*_3_^2^, *M*)∧(*a*_4_^2^, *M*)	(*a*_4_^2^, *M*)
*t*_4_^2^ = (*a*_1_^2^, *F*)∧(*a*_2_^2^, *F*)∧(*a*_3_^2^, *M*)∧(*a*_4_^2^, *M*)	(*a*_1_^2^, *F*)∧(*a*_2_^2^, *F*), (*a*_2_^2^, *F*)∧(*a*_3_^2^, *M*), (*a*_4_^2^, *M*)
*t*_5_^2^ = (*a*_1_^2^, *F*)∧(*a*_2_^2^, *F*)∧(*a*_3_^2^, *M*)∧(*a*_4_^2^, *L*)	(*a*_1_^2^, *F*)∧(*a*_2_^2^, *F*), (*a*_2_^2^, *F*)∧(*a*_3_^2^, *M*), (*a*_2_^2^, *F*)∧(*a*_4_^2^, *L*)
*t*_6_^2^ = (*a*_1_^2^, *F*)∧(*a*_2_^2^, *B*)∧(*a*_3_^2^, *M*)∧(*a*_4_^2^, *M*)	(*a*_1_^2^, *F*)∧(*a*_2_^2^, *B*), (*a*_2_^2^, *B*)∧(*a*_3_^2^, *M*), (*a*_4_^2^, *M*)
*t*_9_^2^ = (*a*_1_^2^, *B*)∧(*a*_2_^2^, *B*)∧(*a*_3_^2^, *H*)∧(*a*_4_^2^, *H*)	(*a*_1_^2^, *B*), (*a*_4_^2^, *H*), (*a*_2_^2^, *B*)∧(*a*_3_^2^, *H*)
*t*_10_^2^ = (*a*_1_^2^, *B*)∧(*a*_2_^2^, *F*)∧(*a*_3_^2^, *H*)∧(*a*_4_^2^, *H*)	(*a*_1_^2^, *B*), (*a*_4_^2^, *H*), (*a*_2_^2^, *F*)∧(*a*_3_^2^, *H*)
*t*_11_^2^ = (*a*_1_^2^, *B*)∧(*a*_2_^2^, *G*)∧(*a*_3_^2^, *H*)∧(*a*_4_^2^, *H*)	(*a*_1_^2^, *B*), (*a*_4_^2^, *H*)
*t*_4_^3^ = (*a*_1_^3^, *N*)∧(*a*_2_^3^, *N*)∧(*a*_3_^3^, *N*)∧(*a*_4_^3^, *Y*)	(*a*_2_^3^, *N*)∧(*a*_4_^3^, *Y*)

**Table 6:** Boundary region reduct descriptors.

Original descriptors	Lower approximation reduct descriptors
*t*_1_^1^ = (*a*_1_^1^, 1)∧(*a*_2_^1^, 1)∧(*a*_3_^1^, 1)∧(*a*_4_^1^, 1)	(*a*_1_^1^, 1)∧(*a*_3_^1^, 1), (*a*_2_^1^, 1)∧(*a*_3_^1^, 1)
*t*_5_^1^ = (*a*_1_^1^, 2)∧(*a*_2_^1^, 2)∧(*a*_3_^1^, 1)∧(*a*_4_^1^, 1)	(*a*_1_^1^, 2)∧(*a*_2_^1^, 2), (*a*_1_^1^, 2)∧(*a*_3_^1^, 1), (*a*_2_^1^, 2)∧(*a*_3_^1^, 1)
*t*_7_^1^ = (*a*_1_^1^, 3)∧(*a*_2_^1^, 3)∧(*a*_3_^1^, 2)∧(*a*_4_^1^, 2)	(*a*_1_^1^, 3)∧(*a*_2_^1^, 3), (*a*_3_^1^, 2), (*a*_1_^1^, 3)∧(*a*_4_^1^, 2)
*t*_9_^1^ = (*a*_1_^1^, 4)∧(*a*_2_^1^, 1)∧(*a*_3_^1^, 3)∧(*a*_4_^1^, 1)	(*a*_1_^1^, 4), (*a*_3_^1^, 3)
*t*_14_^1^ = (*a*_1_^1^, 5)∧(*a*_2_^1^, 2)∧(*a*_3_^1^, 4)∧(*a*_4_^1^, 1)	(*a*_1_^1^, 5), (*a*_3_^1^, 4)
*t*_19_^1^ = (*a*_1_^1^, 6)∧(*a*_2_^1^, 3)∧(*a*_3_^1^, 5)∧(*a*_4_^1^, 2)	(*a*_1_^1^, 6), (*a*_3_^1^, 5)
*t*_1_^2^ = (*a*_1_^2^, *G*)∧(*a*_2_^2^, *G*)∧(*a*_3_^2^, *L*)∧(*a*_4_^2^, *L*)	(*a*_1_^2^, *G*), (*a*_3_^2^, *L*)
*t*_3_^2^ = (*a*_1_^2^, *F*)∧(*a*_2_^2^, *G*)∧(*a*_3_^2^, *M*)∧(*a*_4_^2^, *L*)	(*a*_1_^2^, *F*), (*a*_3_^2^, *M*)
*t*_1_^3^ = (*a*_1_^3^, *Y*)∧(*a*_2_^3^, *Y*)∧(*a*_3_^3^, *Y*)∧(*a*_4_^3^, *Y*)	(*a*_1_^3^, *Y*), (*a*_3_^3^, *Y*)
*t*_2_^3^ = (*a*_1_^3^, *N*)∧(*a*_2_^3^, *Y*)∧(*a*_3_^3^, *N*)∧(*a*_4_^3^, *Y*)	(*a*_1_^3^, *N*)∧(*a*_2_^3^, *Y*), (*a*_1_^3^, *N*)∧(*a*_4_^3^, *Y*), (*a*_2_^3^, *Y*)∧(*a*_3_^3^, *N*), (*a*_3_^3^, *N*)∧(*a*_4_^3^, *Y*)
*t*_3_^3^ = (*a*_1_^3^, *N*)∧(*a*_2_^3^, *N*)∧(*a*_3_^3^, *N*)∧(*a*_4_^3^, *N*)	(*a*_2_^3^, *N*), (*a*_4_^3^, *N*)
